# Supplementary material for: Segmented filamentous bacteria undergo a structural transition at their adhesive tip during unicellular to filament development
Source: Nat Commun. 2025 Dec 17;17:222. doi: 10.1038/s41467-025-66892-5 (PMC12780176; doi:10.1038/s41467-025-66892-5)
Supplement: Supplementary file 1 — Supplementary Information [file 41467_2025_66892_MOESM1_ESM.pdf]

Supplementary Table 1

Supplementary Table 1. Characteristics of vesicles, intracellular filaments and plate-like structures identified at the SFB tip, proximal cell body region or near the SFB tip. Source data are provided as a Source Data file.

SFB origin	Feature	No. SFB analysed	No. features analysed	Diameter[a]/Width[b] (nm)		
				Median	Mean	Standard deviation
Mouse-SFB	Filaments	11[c]	23	8	9	1
	Plate-like structures	3	6	4	4	1
	Intracellular vesicles	10[c]	15	51	54	15
	Extracellular vesicles	7[c]	15	87	85	40
Rat-SFB	Filaments	7[c]	9	9	9	1
	Plate-like structures	2	4	5	6	1
	Intracellular vesicles	5[c]	6	41	62	53
	Extracellular vesicles	12[c]	26	103	103	54

[a] Diameter: measurement for intracellular and extracellular vesicles. SFB with a broken tip were not included in the intracellular vesicle analysis.
[b] Width: measurement for filaments and plate-like structures.
[c] Data from at least two biological replicates.

Supplementary Table 2

Supplementary Table 2. Binding affinity between the VHH anti-Th17Ag and Th17Ag determined Biolayer Interferometry (BLI). Source data are provided as a Source Data file.

Association rate constant (k_{on})	Dissociation rate constant (k_{off})	Equilibrium dissociation constant (K_D)
7.29×10^3 M^{-1} s^{-1}	2.51×10^{-4} s^{-1}	39 nM

Supplementary Table 3

Supplementary Table 3. HDX-MS summary table.

**HDX EXPERIMENT**	**EPITOPE MAPPING**	
Data Set	Apo state (Control)	VHH-bound state
HDX reaction details		
Labelling buffer :	PBS 1X, pD 7.45	PBS 1X, pD 7.45
Temperature:	23°C	23°C
Deuterium excess:	90%	90%
Molar Excess VHH	N/A	1.5X
% Complex during labelling#	N/A	> 90%
HDX time course analyzed (min)	0.16, 1, 10, 30, 60, and 120	0.16, 1, 10, 30, 60, and 120
Number of peptides	154	154
Sequence coverage*	90.6%	90.6%
Average peptide length	15.21	15.21
Redundancy	2.44	2.44
Average peptide length / Redundancy ratio	6.23	6.23
Replicates	2 technical replicates & 1 biological replicate	
Repeatability (pooled standard deviation)	0.090 Da	0.092 Da
Significant difference between state^	Two-sided Wald test, $p < 0.05$	

#considering a *K*d of 39 nM and a 1:1 binding stoichiometry
*sequence coverage after labelling with 1.5X molar excess VHH
^MEMHDX software[1]
N/A: not applicable; HDX: Hydrogen/Deuterium eXchange

Supplementary Table 4

Supplementary Table 4. Primers used to amplify DNA sequences encoding anti-Th17Ag nanobodies.

PCR1	Pair 1	5’ GAT GTG CAG CTG CAG GCG TCT GGR GGA GG 3’<br>5’ CGC CAT CAA GGT ACC AGT TGA 3’
	Pair 2	5’- GGTGGTCCTGGCTGC -3’<br>5’- ATGGAGAGGACGTCCTTGGGT-3’
	Pair 3	5’- GTCCTGGCTGCTCTCTACAAGG-3’<br>5’- GGTACGTGCTGTTGAACTGTTCC -3’
	Pair 4	5’ G GTG GTC CTG GCT GCN CT 3’<br>5’ ATG GAG AGG ACG TCC TTG GGT 3’
	Pair 5	5’ GTC CTG GCT GCT CTW YTA CAR GG 3’<br>5’ GGT ACG TGC TGT TGA ACT GTT CC 3’
PCR2	Pair 6	5’ CATGCCATGACTCGCGGCCCAGCCGGCCATGGCCGAKGTSCAGCT 3’<br>5’ GGACTAGTTGCGGCCGCTGAGGAGACGGTGACCTG 3’
	Pair 7	5’ GTCATTGGCCCAGCCGGCCATGGCTCAGKTGCAGCTCGTGGAGTCNGG 3’<br>5’ GACATTGCGGCCGCGCTGGGGTCTTCGCTGTGGTG 3’
	Pair 8	5’ GTCATTGGCCCAGCCGGCCATGGCTCAGKTGCAGCTCGTGGAGTCNGG 3’<br>5’ GACATTGCGGCCGCTGGTTGTGGTTTTGGTGTCTTGGG 3’

Supplementary Fig. 1

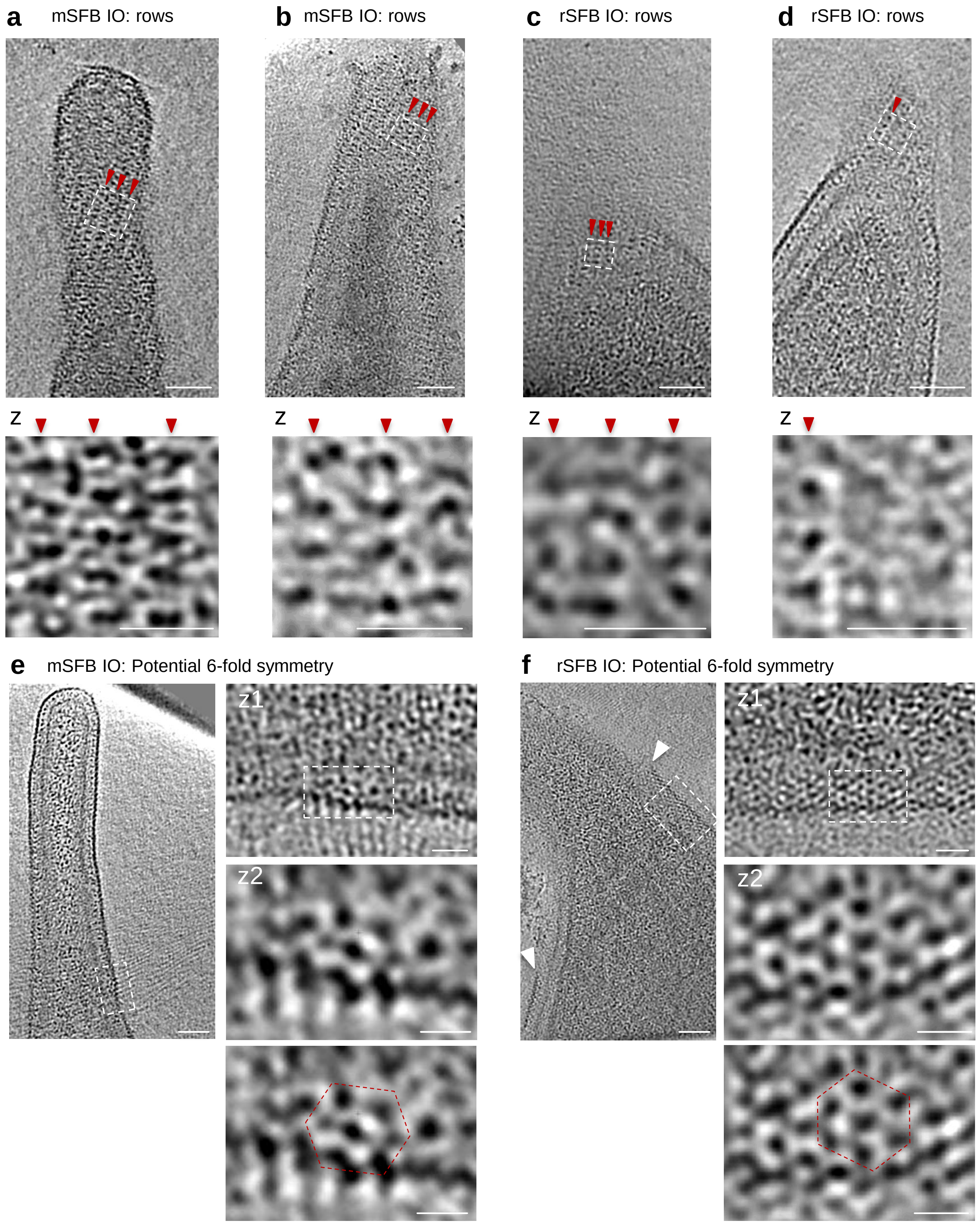

Supplementary Fig. 1. Top view of the S-layer arrangement at the SFB tip. a-c, Top view of the organization of S-layer subunits in rows seen in tomograms from the tip of **(a**,**b)** mouse-SFB (EMD-52685, EMD-52687) and **(c**,**d)** rat-SFB IOs (EMD-52688,EMD-52684). Close-ups (z) of the regions delimited by a white dashed line were included below each panel. Red arrow heads show S-layer subunits arranged in rows. **e**,**f**, Top view of tomograms which include the tip of **(e)** mouse-SFB (EMD-52655) and **(f)** rat-SFB IOs (EMD-52689), showing a potential six-fold symmetry of the S-layer subunits. Close-ups (z) of the regions delimited by a white dashed line were included below or next to each panel. z2 panels were duplicated to highlight example regions of a potential 6-fold symmetry (red dashed hexagons). S-layer discontinuities at the SFB tip resulting in a broken tip phenotype are shown by white arrow heads. mSFB: mouse-SFB, rSFB: rat-SFB. **Scale bars**: a-f: 50 nm; a-f(z): 20 nm.

Supplementary Fig. 2

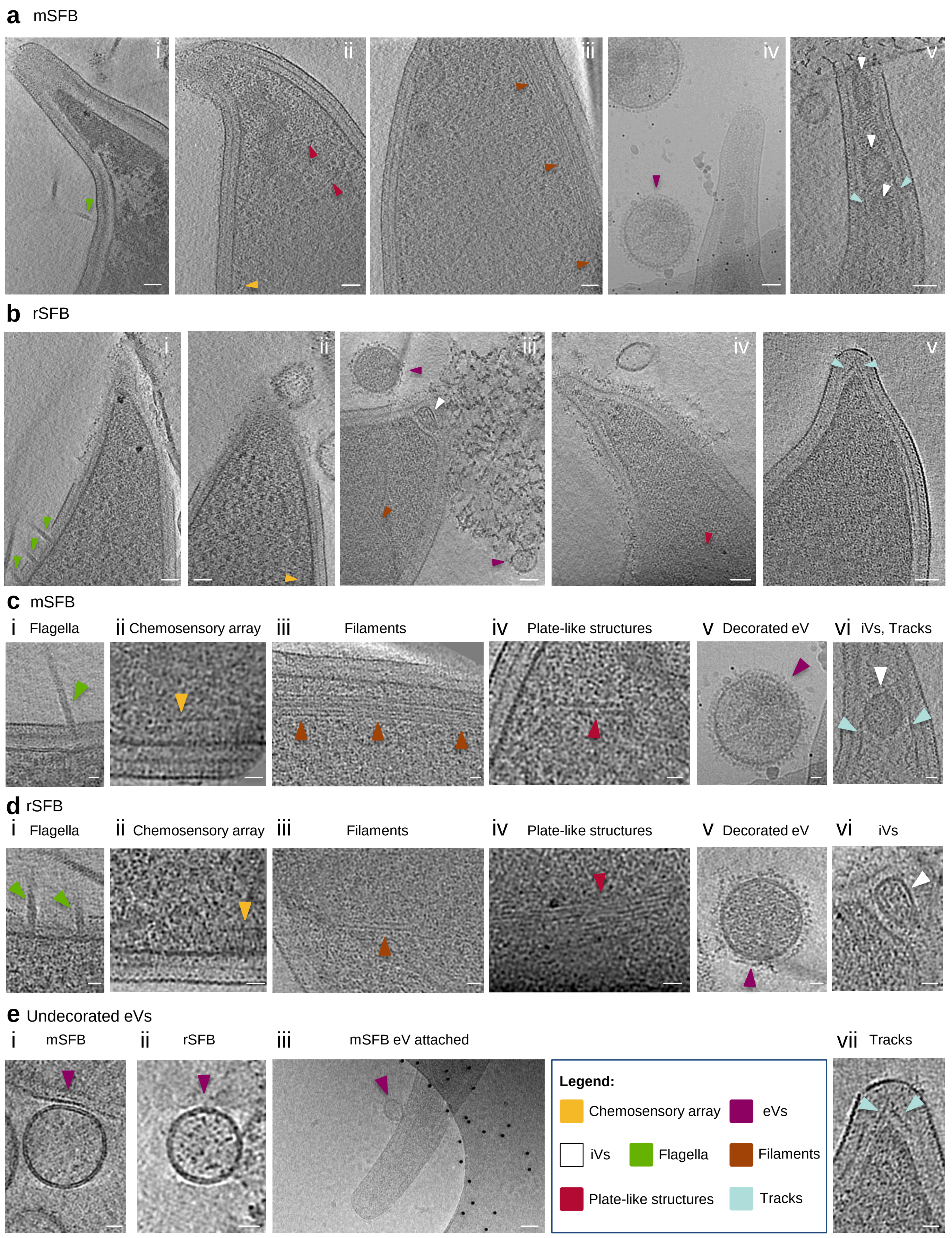

Supplementary Fig. 2. Additional examples of the intracellular and extracellular features identified at or near the SFB tip and proximal region of the cell body of SFB; Fig. 2. a(i-iii,**v)**, Representative tomographic slices from mouse-SFB IOs tomograms EMD-52687, EMD-52673, EMD-52674 and EMD-52670 containing intracellular and extracellular features. The corresponding IOs had the following length: 2.5, 2.3, 2.5, 2.1, 2.5 µm. **a(iv)**, Representative projection image from mouse-SFB containing decorated extracellular vesicles. **b(i-ii)**, representative tomographic slices showing **(b(i))** flagella and **(b(ii))** a chemosensory array present in an IO with 2.2 µm in length (EMD-52692). **b(iii-v)**, Representative tomographic slices from rat-SFB IOs tomograms EMD-52693, EMD-52694 and EMD-52695 showing **(b(iii))** filaments, iVs and eVs **(b(iv))** plate-like structures and **(b(v))** tracks. The corresponding IOs had the following length: 2.0, 2.0 and 2.2 µm. **c/d**, Close-ups from the tomographic slices of **(c)** mouse-SFB and **(d)** rat-SFB shown in a and b, respectively, that contain the following features: **(c-d(i))** flagella, **(c-d(ii))** chemosensory array, **(c-d(iii))** filaments, **(c-d(iv))** plate-like structures, **(c-d(v))** decorated extracellular vesicles (eVs), **(c-d(vi))** intracellular vesicles and **(c(vi)**,**d(vii))** tracks. **e(i-ii)**, Tomographic slices of undecorated eVs found near **(e(i))** mouse-SFB (EMD-52675) and **(e(ii))** rat-SFB IOs (EMD-52693). **e(iii)**, Projection image containing an extracellular vesicle in direct contact with the tip of a mouse-SFB IO. The image was acquired with a Tecnai F20 electron microscope equipped with a Falcon 2 camera. The identified features are shown by arrow heads of the colors indicated in the legend. mSFB: mouse-SFB, rSFB: rat-SFB, iVs: intracellular vesicles, eVs: extracellular vesicles. **Scale bars**: a-b, e(iii): 50 nm; c/d, e(i-ii): 20 nm.

Supplementary Fig. 3

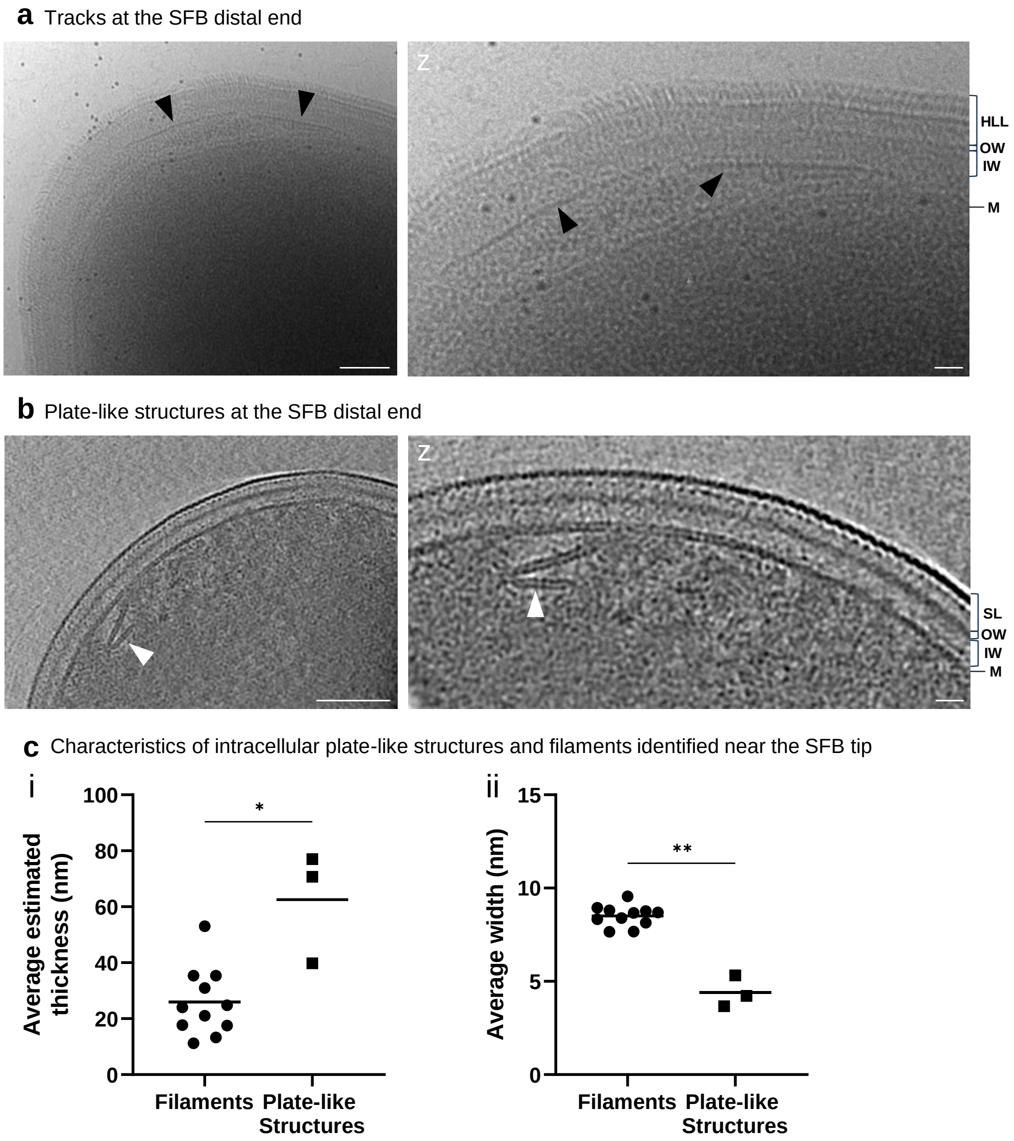

Supplementary Fig. 3. Description of intracellular features at the mouse-SFB distal end and further characterization near the mouse-SFB tip. a, Representative projection image and **(z)** corresponding close-up showing tracks (black arrow heads) identified in the distal end of mouse-SFB. **b**, Representative tomographic slice of the tomogram EMD-52696 and **(z)** corresponding close-up showing plate-like structures (white arrow heads) identified in the distal end of mouse-SFB. HLL: hair-like layer; SL: Surface layer (S-layer), OW: outer wall zone, IW: inner wall zone, M: membrane. **c**, Measurements of the **(c(i))** estimated thickness and **(c(ii))** width for intracellular filaments (n=11 filaments from 2 independent experiments) and plate-like structures (n=3 plate-like structures from 1 experiment) identified near the tip of mouse-SFB. The average of 10 measurements performed in different regions was plotted for the tracks found in each SFB analyzed and the corresponding mean is shown. The statistical significance was assessed using a two-sided Mann–Whitney U test (c(i): p = 0.0110; c(ii): p = 0.0055). Source data are provided as a Source Data file. **Scale bars**: a-b: 100 nm; z: 20 nm.

Supplementary Fig. 4

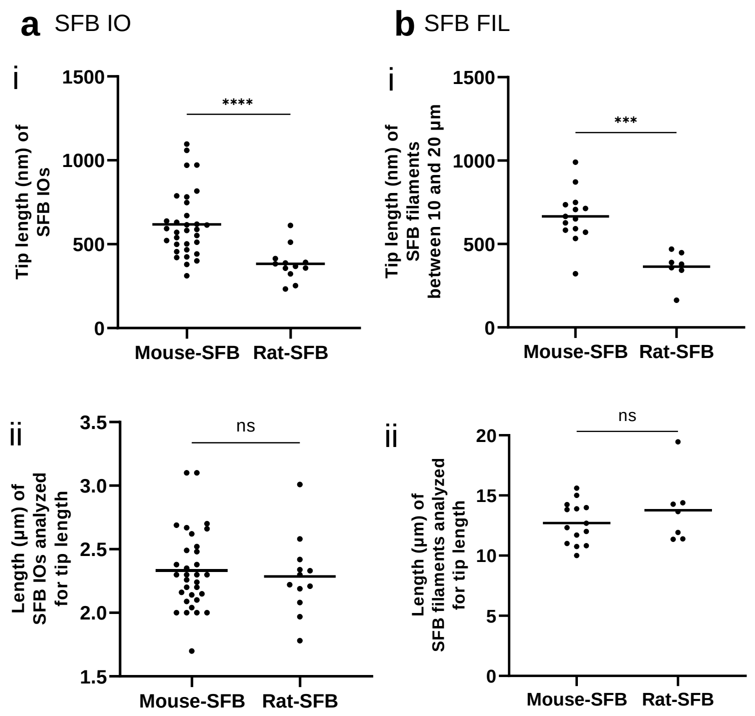

Supplementary Fig. 4. Differences in tip length between mouse-SFB and rat-SFB. a,**b(i)**, Tip length of mouse-SFB and rat-SFB **(a(i))** IOs and **(b(i))** filaments (FIL). **a**,**b(ii)**, Length of mouse-SFB and rat-SFB **(a(ii))** IOs and **(b(ii))** filaments included in the tip length analysis. Only IOs assigned to Stages 1-3 were included in the analysis (n=32 mouse-SFB IOs from 6 independent experiments and n=12 rat-SFB IOs from 4 independent experiments) of a(ii). Only filaments (n=14 mouse-SFB filaments from 5 independent experiments and n=7 rat-SFB filaments from 3 independent experiments) with a length between 10 and 20 µm were included in the analysis of b(ii). Individual measurements and the corresponding mean are shown. The statistical significance was assessed using a two-sided Mann–Whitney U test (a(i): $p < 0.0001$, b(i): $p = 0.0005$, a,b(ii): ns: not significant). Source data are provided as a Source Data file for all panels.

Supplementary Fig. 5

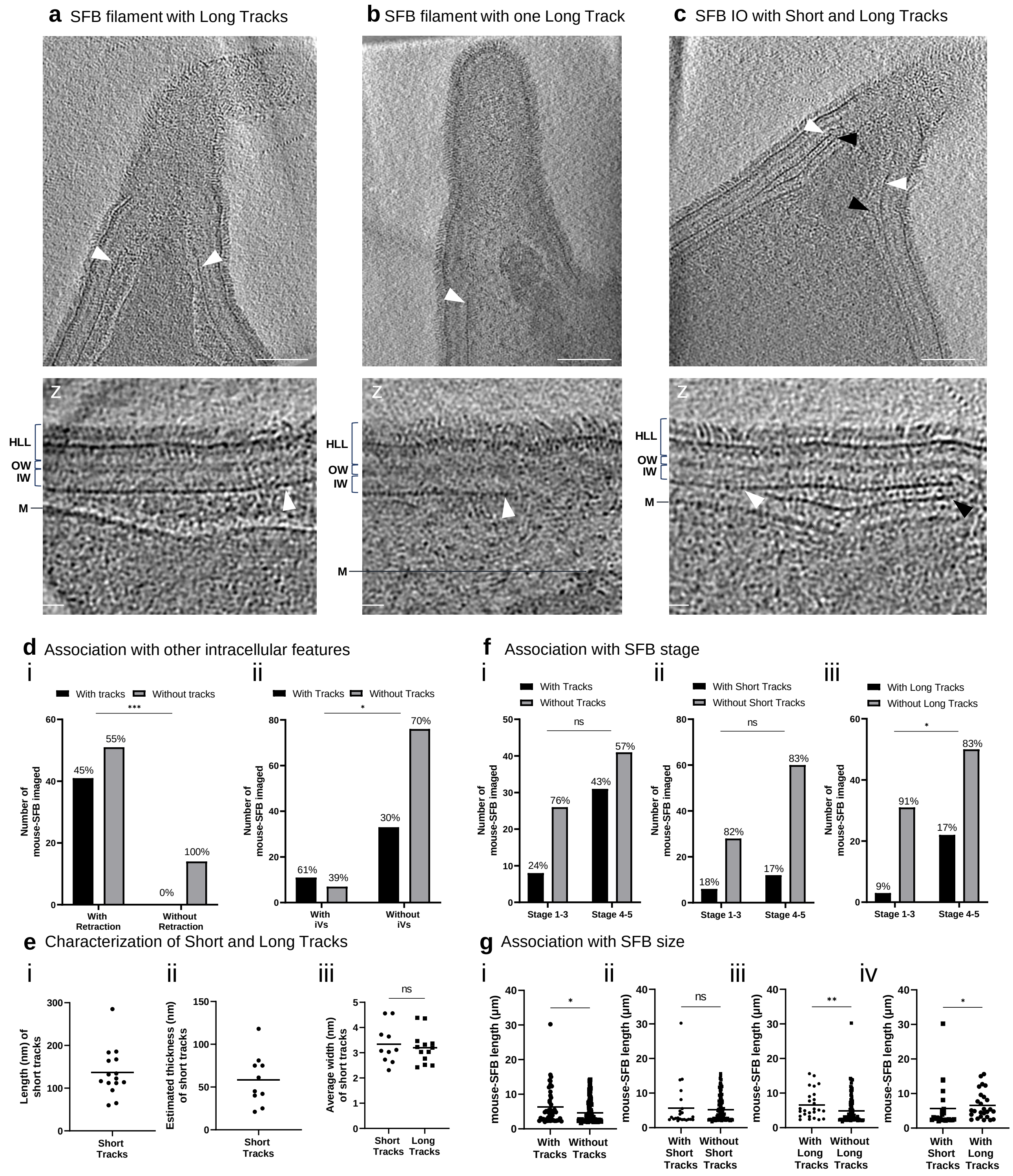

Supplementary Fig. 5. Characterization of mouse-SFB tracks. a-c, Representative tomographic slices showing Stage 4/5 SFB containing **(a)** two long tracks (EMD-52677), **(b)** one long track (EMD-52678) and **(c)** both short and long tracks (EMD-54605). Long and short tracks are shown by white and black arrow heads, respectively. **a-c(z)**, Close-ups of the regions containing tracks are shown. HLL: hair-like layer; OW: outer wall zone; IW: inner wall zone; M: membrane. **d**, Comparison of the proportions of mouse-SFB with and without **(d(i))** membrane retraction (n=92 SFB with and n=14 SFB without membrane retraction from 7 and 4 independent experiments, respectively) or **(d(ii))** intracellular vesicles (iVs) in which tracks were identified (n=18 SFB with and n=110 SFB without iVs from 4 and 7 independent experiments, respectively). **e**, Measurements of the **(e(i))** length and **(e(ii))** estimated thickness for short tracks and **(e(iii))** width for both short (n=10 SFB from one experiment) and long tracks (n=13 SFB from 3 independent experiments) found in mouse-SFB. The average of 10 measurements performed in different regions is shown for the tracks found in each SFB analyzed. **f**, Comparison of the proportions of mouse-SFB from early (Stages 1-3) and late stages (Stages 4-5) in which **(f(i))** any tracks (n=39 SFB from 6 independent experiments), **(f(ii))** short tracks (n=18 SFB from 4 independent experiments) and **(f(iii))** long tracks (n=25 SFB from 6 independent experiments) were identified. **g(i-iii)**, Assessment of an association of mouse-SFB length with the presence of **(g(i))** any tracks (n=44 SFB from 6 independent experiments), **(g(ii))** short tracks (n=23 SFB from 5 independent experiments) and **(g(iii))** long tracks (n=26 SFB from 6 independent experiments). **g(iv)**, Comparison between the length of mouse-SFB in which short or long tracks were identified. The statistical significance was assessed for panels d/f using a two-sided Fisher's exact test (d(i): $p = 0.0007$; d(ii): $p = 0.0157$; f(iii): $p = 0.0146$; ns: not significant), for panels e(i) using a two-sided unpaired t-test and for panels g(i-iii) using a two-sided Mann–Whitney U test (g(i): $p = 0.0431$; g(iii): $p = 0.0036$; g(iv): $p = 0.0331$). For panels e/g, the mean is shown. Source data are provided as a Source Data file for panels d-g. **Scale bars**: a-c: 100 nm; z: 20 nm.

Supplementary Fig. 6

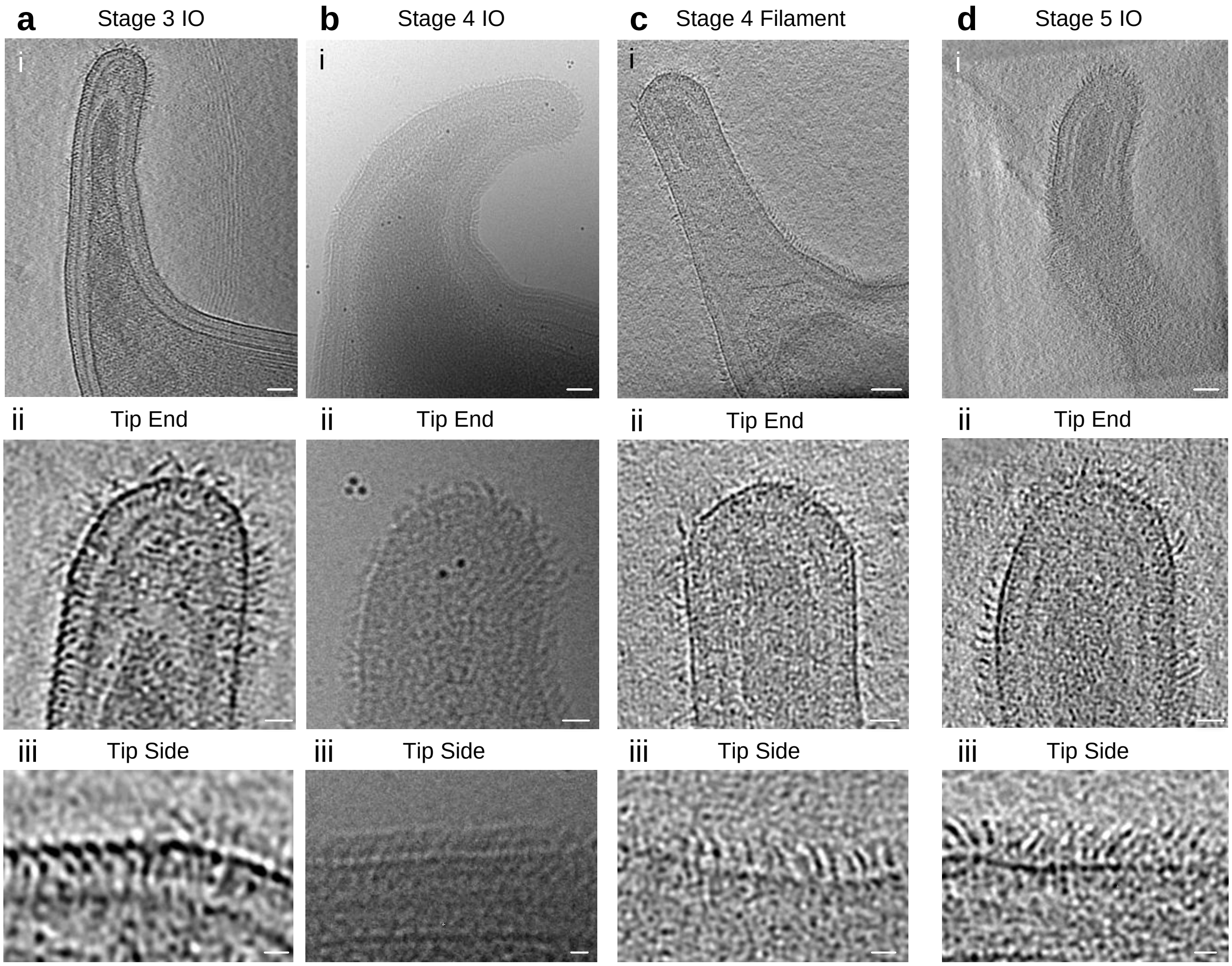

Supplementary Fig. 6. Additional examples of the developmental stages of mouse-SFB; related to Fig. 3. a-d, Representative (**a**,**c**,**d**) tomographic slices from reconstructed tomograms EMD-52680, EMD-54608 and EMD-52682 and **(b)** projection image showing the tip of mouse-SFB assigned to Stages: **(a(i))** 3, **(b-c(i))** 4 and **(d(i))** 5. The selected SFB had a length of 2.3, 4.7, 9.4 and 4.8 µm, respectively. **a-c(ii-iii)**, Close-ups of the **(a-d(ii))** IOs tip end and **(a-d(iii))** tip side from Stages 3, 4 and 5. **Scale bars**: a-d(i): 50 nm; a-d(ii,iii): 20 nm.

Supplementary Fig. 7

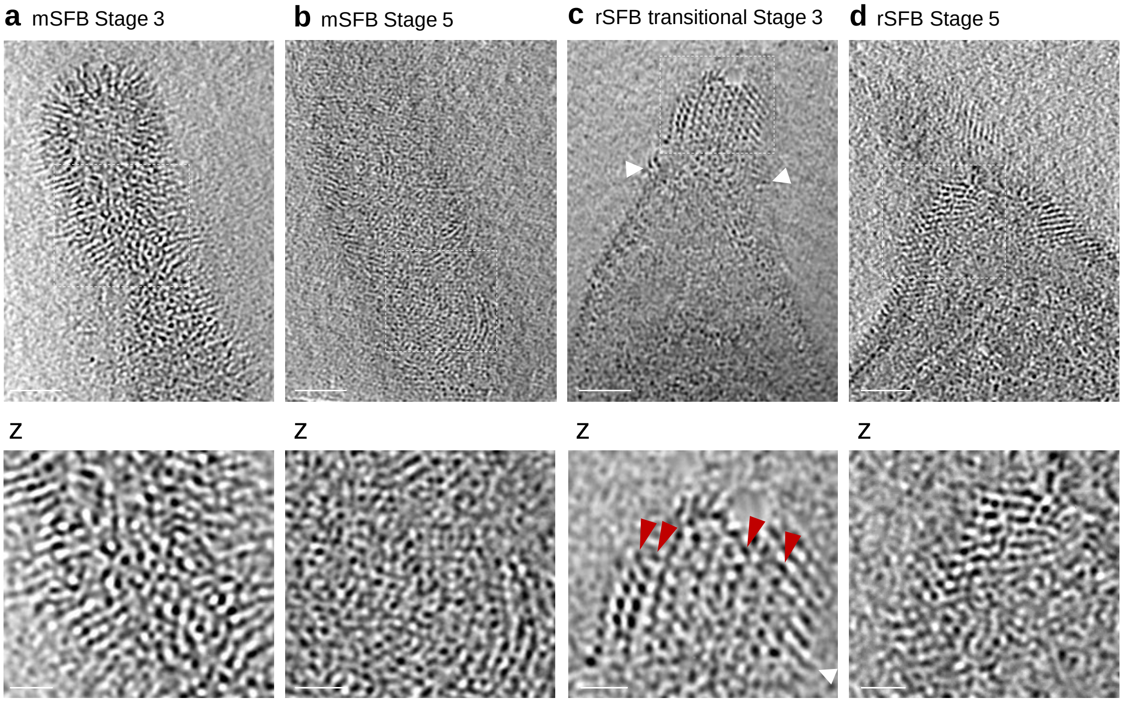

Supplementary Fig. 7. Top view of different stages of the SFB tip. a/b, Representative tomographic slices of the mouse-SFB tip showing the top view of: **(a)** disordered hair-like structures and **(b)** hair-like layer (HLL) subunits present in Stage 3 (EMD-52676) and Stage 5 (EMD-52678) SFB, respectively. **c/d**, Representative tomographic slices of the rat-SFB tip showing the top view of: **(c)** the HLL subunits arrangement in a potential transitional stage from Stage 3 to Stage 4 (EMD-54603) and **(d)** the HLL subunits present in Stage 5 SFB (EMD-54607). Red arrow heads shows a potential arrangement of the HLL subunits in rows (c). White arrow heads indicate the region until which the HLL can be seen for the transitional Stage 3 in c and an example of a hair-like structure in c(z). Close-ups **(z)** of the regions delimited by a white dashed line were included below each panel. mSFB: mouse-SFB, rSFB: rat-SFB. **Scale bars**: a-d**:** 50 nm; a-d(z): 20 nm.

Supplementary Fig. 8

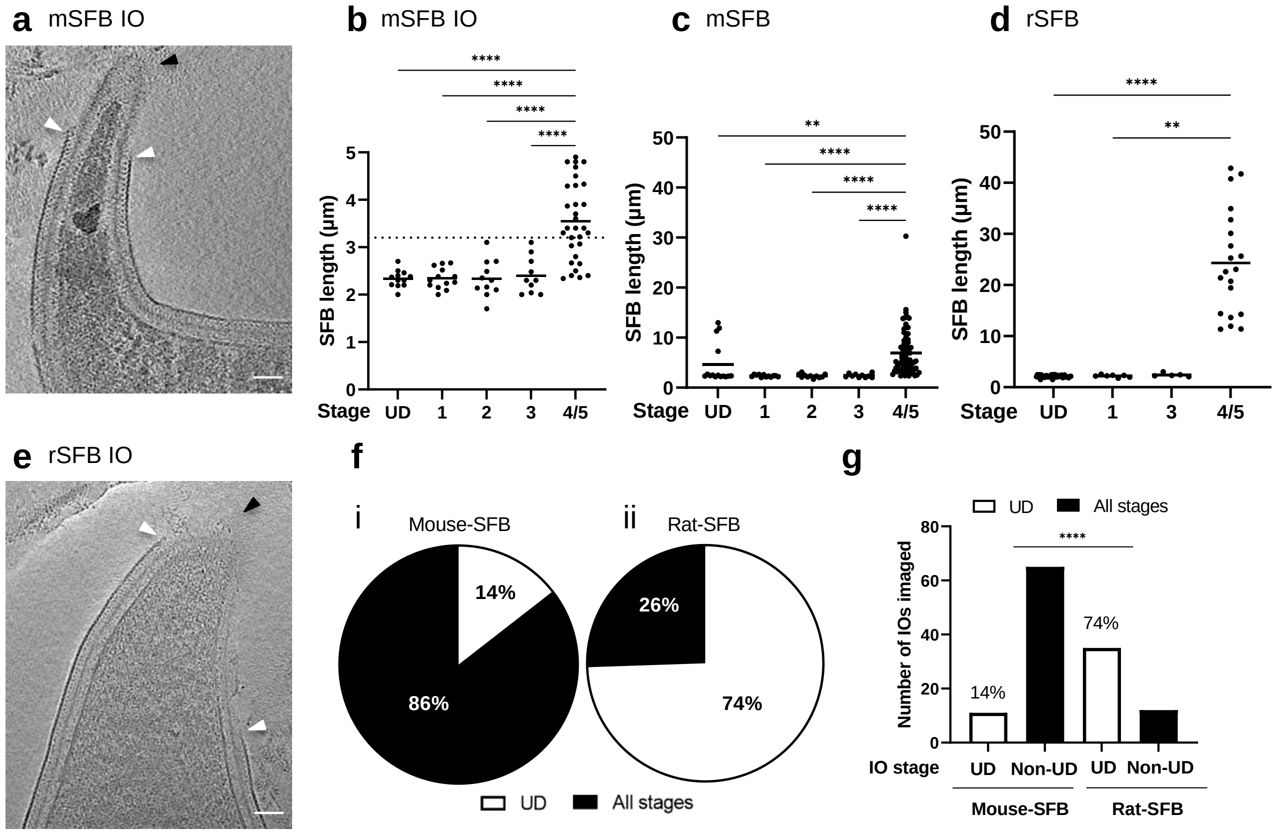

Supplementary Fig. 8. Characterization of unidentified SFB stages. a/e, Tomographic slices of representative **(a)** mouse-SFB (EMD-52697) and **(e)** rat-SFB IOs (EMD-52690) that could not be assigned to a tip stage (UD: unidentified). Discontinuities in the IOs S-layer are shown by white arrow heads. The absence of disHLS at the tip end is shown by black arrow heads. **b-d**, Length of: **(b)** mouse-SFB IOs grown in mice, **(c)** total mouse-SFB grown in mice and **(d)** total rat-SFB grown in rats assigned to each tip stage. All SFB imaged were included in the analysis even if no distinction between Stage 4 and Stage 5 could be made (4/5) and if the tip stage could not be identified (UD, undetermined) (a: n=123, 7 independent experiments; b: n=77, 7 independent experiments; c: n=63, 5 independent experiments; d: n=79, 4 independent experiments). Individual measurements and the corresponding mean are shown. For panel a, the statistical significance was assessed using the one-way ANOVA ($p < 0.0001$). A dashed line was included at an IO length of 3.2 µm. For panels b and c, the statistical significance was assessed using the Kruskal-Wallis test followed by a Dunn's test correction for multiple comparisons (b: Stage 4/5 vs Stage1, Stage 2 and Stage 3: $p < 0.0001$, Stage 4/5 vs Stage UD: $p = 0.0077$; c: Stage 1 vs Stage 4/5, $p = 0.0014$; UD vs Stage 4/5, $p < 0.0001$). **f**, Proportion of **(f(i))** mouse-SFB (n=11 IOs from 4 independent experiments) and **(f(ii))** rat-SFB IOs (n=35 IOs from 5 independent experiments) assigned to a tip stage. mSFB: mouse-SFB, rSFB: rat-SFB. **g**, Number of mouse-SFB and rat-SFB IOs in which the tip stage could not be determined due to discontinuities in the S-layer. The statistical significance between the proportion of the tip stages of mouse-SFB and rat-SFB IOs was assessed using a two-sided Fisher's exact test ($p < 0.0001$). Source data are provided as a Source Data file for panels b-d, f-g. The same data were used to prepare panels f and g. **Scale bar**: a/e: 50 nm.

Supplementary Fig. 9

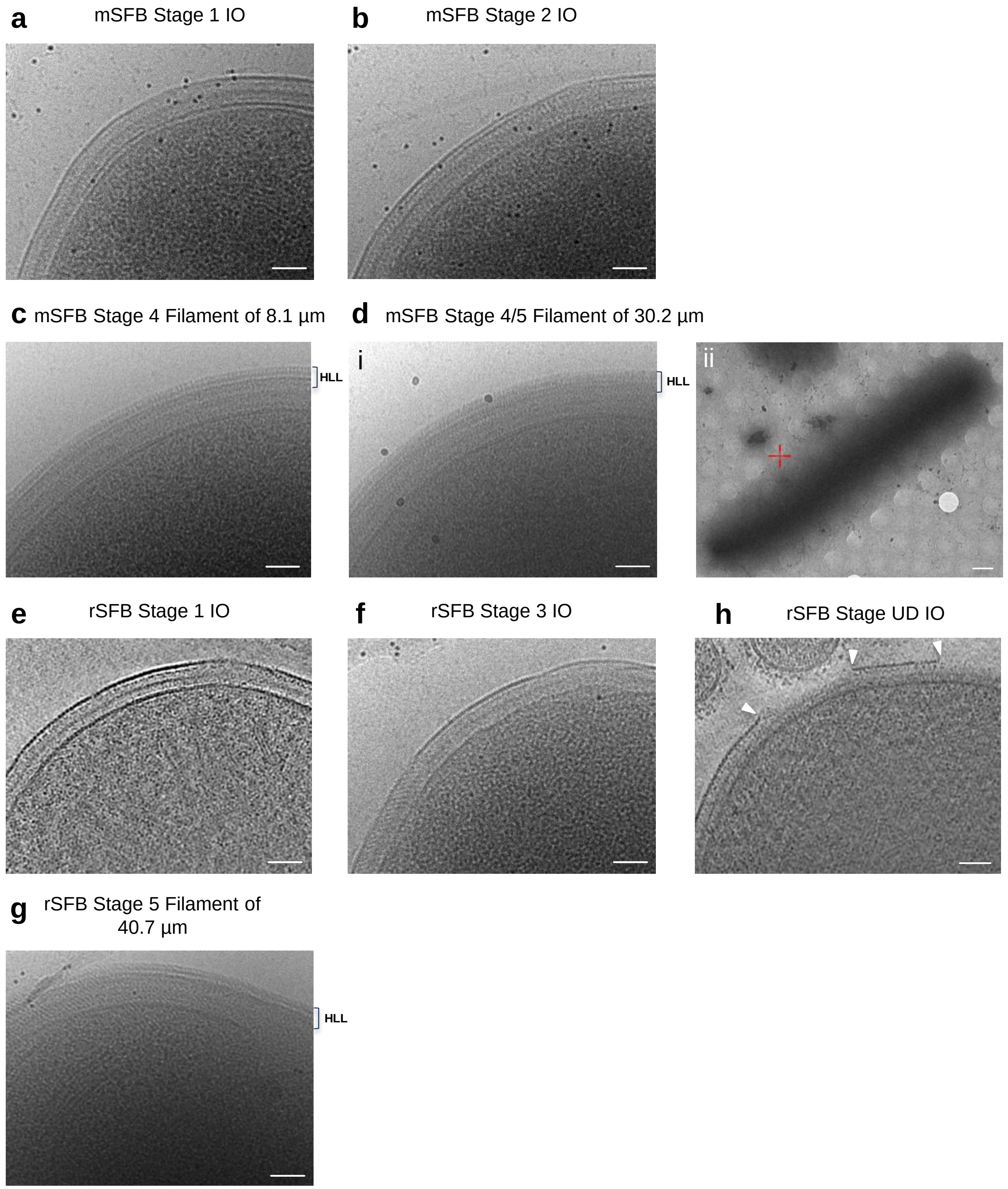

Supplementary Fig. 9. Assessment of the presence of the hair-like layer at the SFB distal end. a-b, Representative projection images of the distal end of mouse-SFB IOs assigned to Stages **(a)** 1 and **(b)** 2. **c-d**, Representative projection images of mouse-SFB filaments assigned to Stages: **(c)** 4 (8.1 µm filament length) and **(d)** 4/5 (30.2 µm filament length). The distal end of mouse-SFB filaments assigned to Stages **(c)** 4 and **(d(i))** 4/5, and **(d(ii))** a long SFB filament (30.2 µm filament length) are shown. **e**, Representative tomographic slice of the distal end of rat-SFB IOs assigned to Stage 1 (EMD-52698). **f**, Representative projection image of the distal end of rat-SFB IOs assigned to Stage 3. **g**, Representative projection image of the distal end of a rat-SFB filament assigned to Stage 5 (40.7 µm filament length). The hair-like layer (HLL) is delimited when present. **h**, Representative tomographic slice from the distal end of a rat-SFB IO with an unidentified stage (UD) displaying S-layer discontinuity also at the distal end (highlighted with white arrow heads) (EMD-52856). **Scale bars**: a-c, d(i), e-h: 50 nm; d(ii): 2 µm.

Supplementary Fig. 10

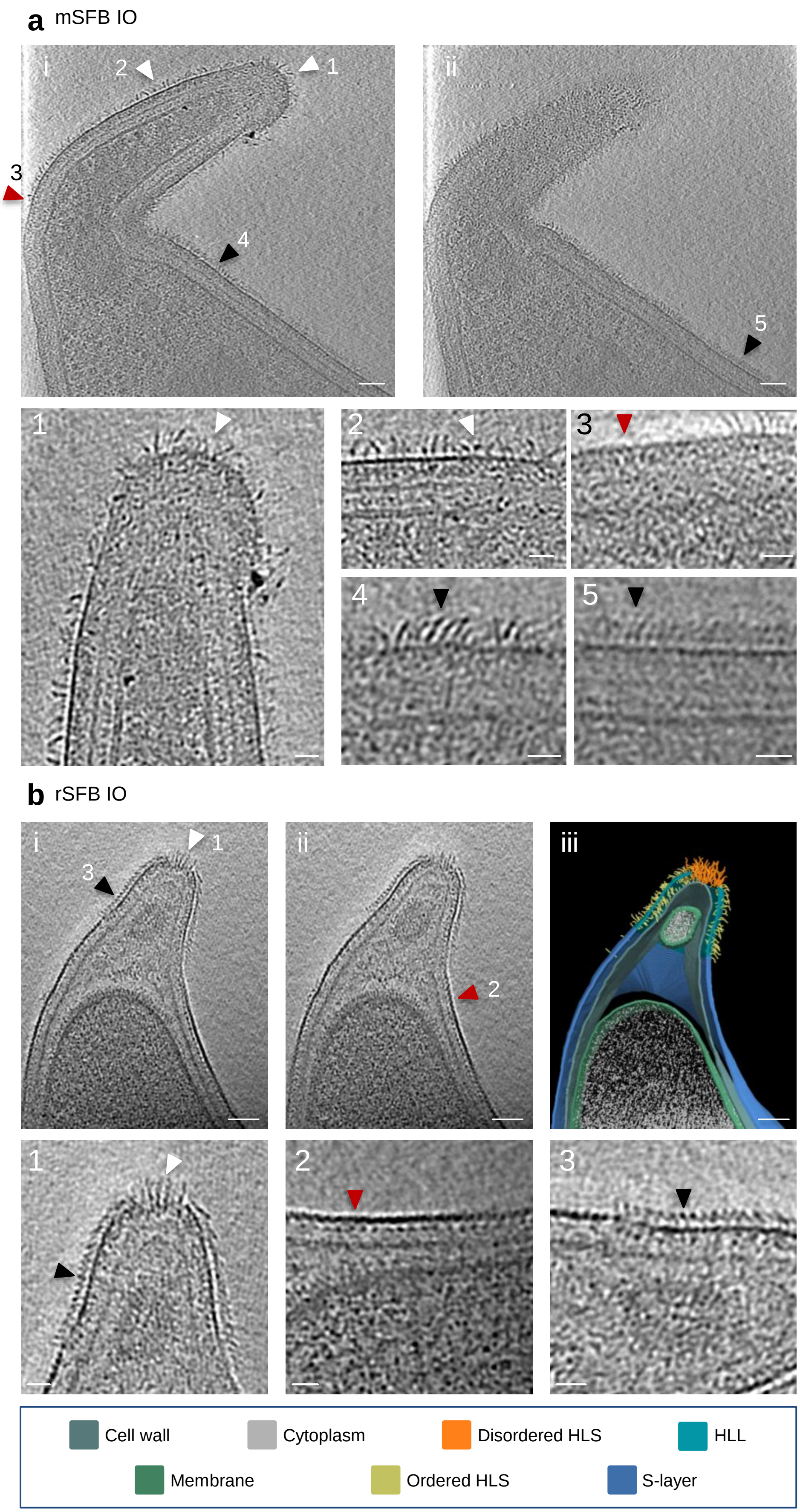

Supplementary Fig. 10. Potential transitional stage between Stage 3 and Stage 4. a-b(i/ii), Tomographic slices at different tilts from **(a(i/ii))** the mouse-SFB IO tomogram EMD-52683 (3.3 µm in length) and **(b(i/ii))** the rat-SFB IO tomogram EMD-54603 (2.2 µm in length) showing the tip and beginning of the cell body. For **(a)** mouse-SFB (mSFB), tomographic slices include: **(1,2)** disordered hair-like structures (white arrow heads), **(3)** regions without HLS (red arrow head), and **(4,5)** a hair-like layer (black arrow heads). For **(b)** rat-SFB (rSFB), tomographic slices include: **(1)** disordered hair-like structures (white arrow heads), **(2)** S-layer-containing regions (red arrow head), and **(3)** small regions with the characteristics of a hair-like layer (black arrow heads). The regions from the which close-ups were taken (1-5) are indicated in panels a-b(i-ii) by arrow heads labelled with the letter of the corresponding panel. **b(iii)**, Segmentation rendering of the rat-SFB tip from tomogram EMD-54603 (Supplementary movie 9). The color legend is displayed at the bottom of the figure. The coloring of the hair-like structures (HLS) denotes their qualitative appearance. Surface layer: S-layer, HLL: hair-like layer. **Scale bars**: a(i-ii),b(i-iii): 50 nm; a(1-5), b(1-3): 20 nm.

Supplementary Fig. 11

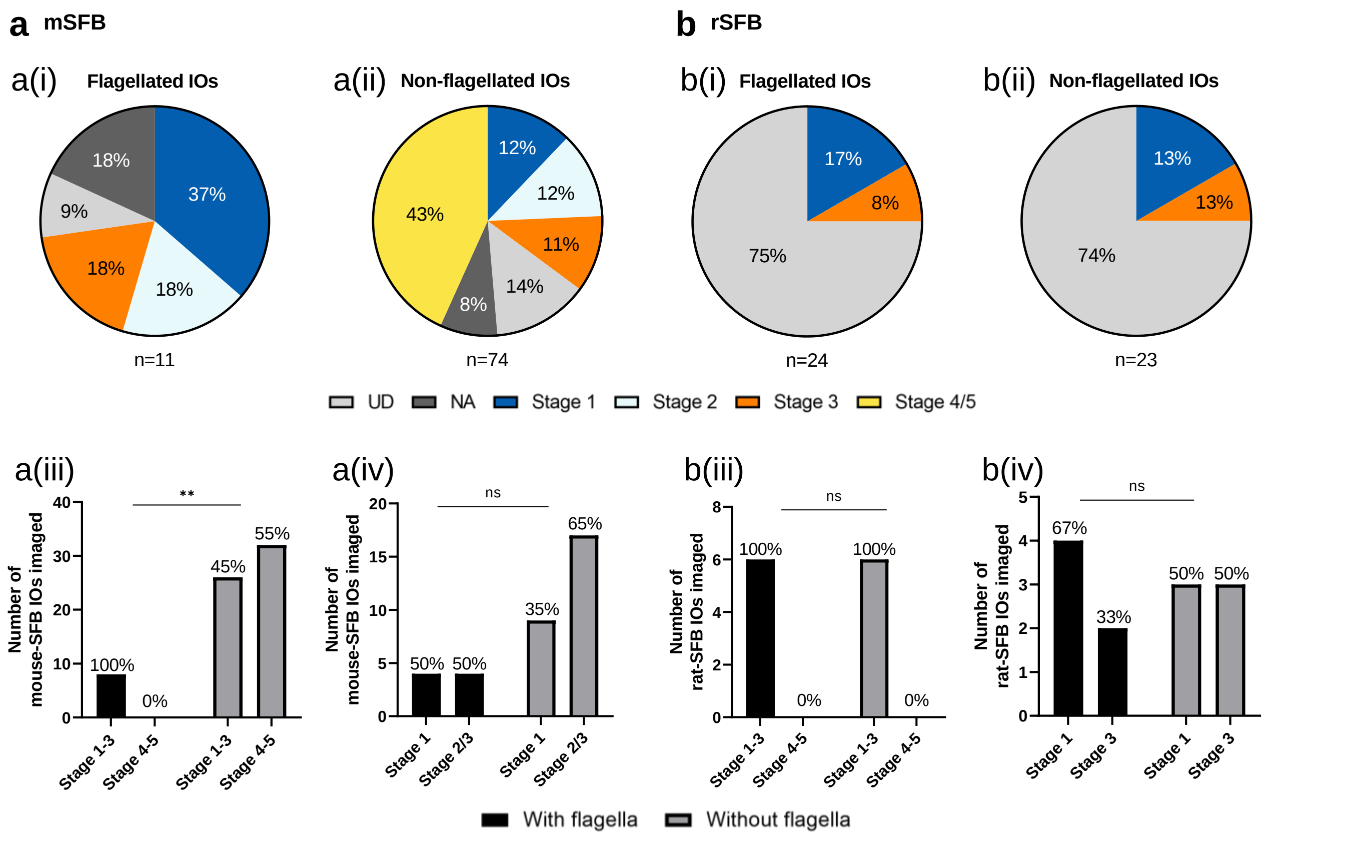

Supplementary Fig. 11. Presence of flagella according to the SFB stage. a/b(i-ii), Pie charts showing the percentage of **(a/b(i))** flagellated and **(a/b(ii))** non-flagellated IOs (≤ 5 µm) of **(a)** mouse-SFB (mSFB, n=11 flagellated and n=74 non-flagellated IOs from 4 and 6 independent experiments, respectively) and **(b)** rat-SFB (rSFB, n=24 flagellated and n=23 non-flagellated IOs from 5 and 4 independent experiments, respectively) identified in each tip stage. **a/b(iii)**, Comparison of the proportions of **(a)** mouse-SFB and **(b)** rat-SFB IOs assigned to early (Stages 1-3) and late SFB stages (Stages 4-5). **a/b(iv)**, Comparison of the proportions of **(a)** mouse-SFB and **(b)** rat-SFB IOs assigned to Stage 1 and other early-SFB stages (Stages 2-3). The statistical significance was assessed using a two-sided Fisher's exact test (a(iii): p = 0.005). Source data are provided as a Source Data file for all panels. The same data were used to prepare the subpanels from panel a. The same data were used to prepare the subpanels from panel b. NA: not assessed, UD: unidentified.

Supplementary Fig. 12

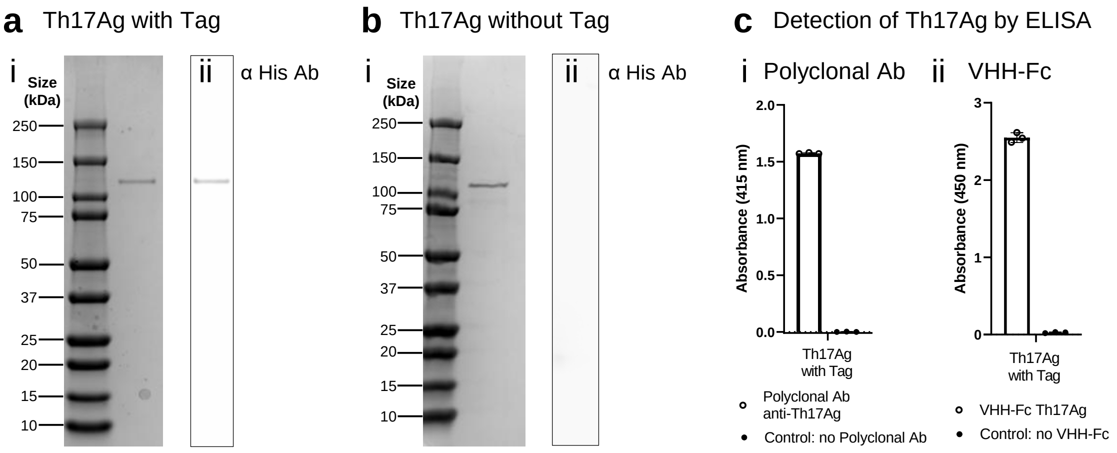

Supplementary Fig. 12. Purification and detection of a Th17 antigen (Th17Ag). a-b(i), SDS-PAGE gel stained with Coomassie brilliant blue R-250 showing the purified Th17Ag expressed in *Escherichia coli* BL21 DE3 Star **(a)** before and **(b)** after the cleavage of the N-terminal 6xHis and V5 epitope-containing tag. **a-b(ii)**, Western blot of the Th17Ag using an anti-polyhistidine antibody (α His Ab) in the conditions described for a-b(i), respectively. A band corresponding to the tagged protein can be seen before the cleavage of the N-terminal tag **(a(ii))** but not after the cleavage of the N-terminal tag **(b(ii))**. Molecular weight of the tagged and untagged protein estimated by Expasy Server[2] is 114 kDa and 110 kDa, respectively. The expression and purification of the Th17Ag was repeated 4 times showing similar results. **c**, Detection by enzyme-linked immunosorbent assay (ELISA) of the purified Th17Ag. Binding was detected using: **(c(i))** a rabbit polyclonal antibody (Ab) and a secondary anti-rabbit antibody conjugated with alkaline phosphatase and **(c(ii))** a nanobody (VHH) coupled to the Fc of the human IgG1 and a secondary antibody anti-Human IgG Fc fragment conjugated with horseradish peroxidase. The mean and standard deviation of three replicates are shown. Source data are provided as a Source Data file for all panels.

Supplementary Fig. 13

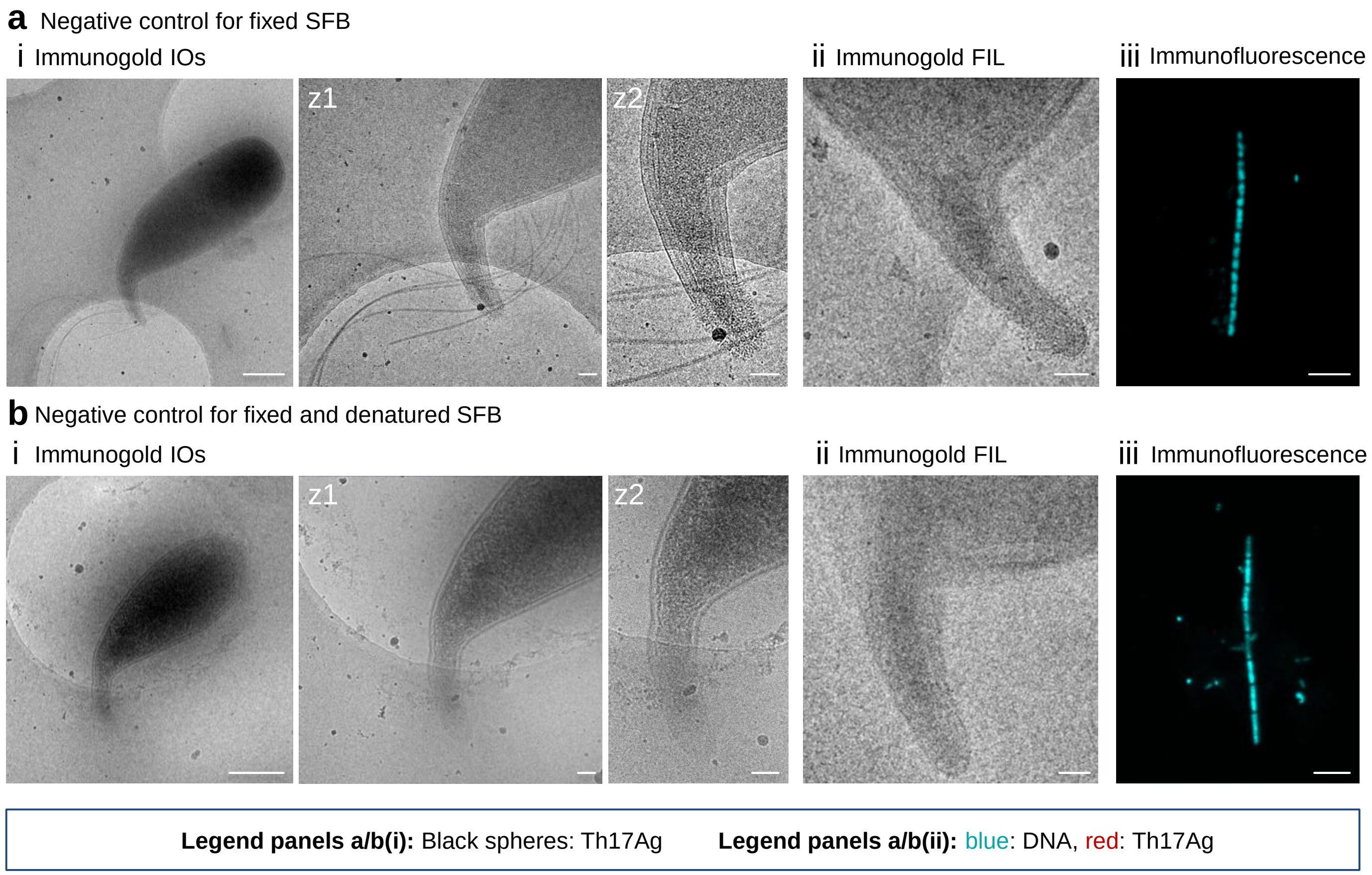

Supplementary Fig. 13. Negative controls of immunofluorescence and immunogold labelling. a, Labelling of fixed SFB with **(a(i-ii))** gold-conjugated Protein A or with **(a(iii))** DAPI and secondary antibody anti-Human IgG Fc Fragment conjugated with Alexa 568. **b**, Labelling of fixed and denatured SFB with **(b(i-ii))** Protein A conjugated with 5 nm gold particles or with **(b(iii))** DAPI and secondary antibody anti-rabbit conjugated with Alexa 568. Projection images of both **(a/b(i))** IOs and **(a/b(ii))** filaments (FIL) are shown. Close-ups (z) are shown for the IOs included in panels a/b(i). Projection images of negative controls for immunogold-labelled SFB were acquired with a Tecnai F20 electron microscope equipped with a Falcon 2 camera. For all immunogold and immunofluorescence experiments, labelling was performed in two distinct days using two biological replicates for each experiment. **Scale bars**: a-b(i): 500 nm; a/b(z,ii): 100 nm; a-b(iii): 5 µm.

Supplementary Fig. 14

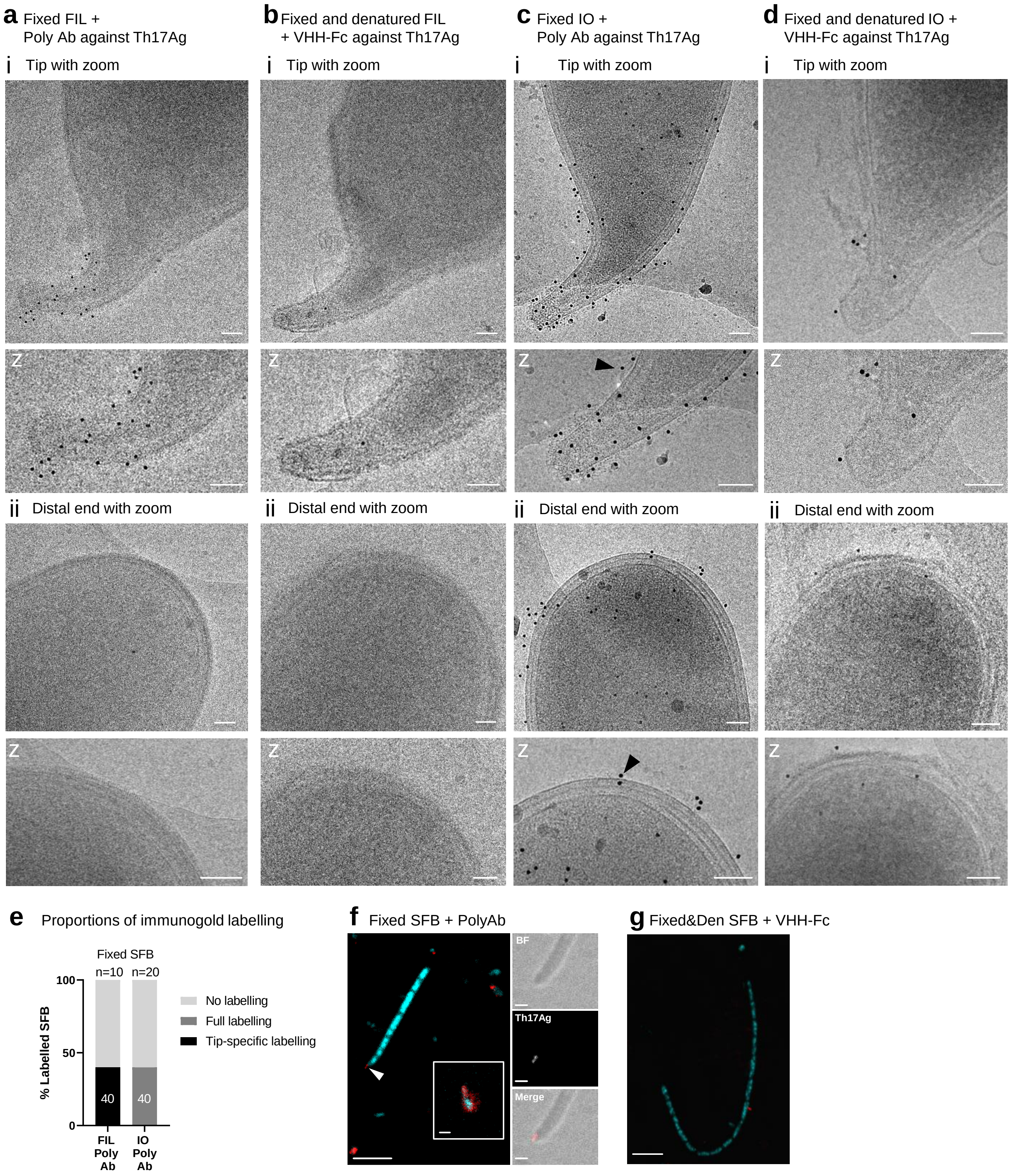

Legend panels a/c: Black spheres: Th17Ag

Legend panels d/e: blue: DNA, red: Th17Ag

Supplementary Fig. 14. Additional data for the localization of the Th17Ag at the SFB surface. a-d, Projection images from purified SFB **(a/b)** filaments (FIL) and **(c/d)** IOs stained using immunogold labelling. Fixed and denatured (Den) SFB **(a)** filaments and **(c)** IOs were incubated with an anti-Th17Ag nanobody fused to the Fc region of human igG1 (VHH-Fc). Fixed SFB **(b)** filaments and **(d)** IOs were incubated with a rabbit polyclonal antibody (Poly Ab) against the Th17Ag. Gold-conjugated Protein A was used for immunogold labelling. Imaging was performed at the SFB **(a-d(i))** tip and **(a-d(ii))** distal end. Close-ups **(z)** of the SFB tip and distal end are shown under each panel. Examples of labelled regions without visible hair-like structures are shown by black arrow heads. Projection images were acquired with a Tecnai F20 electron microscope equipped with a Falcon 2 camera. **e**, Assessment of immunogold labelling for SFB imaged in the condition described for panel d. The percentage of SFB labelled and labelled specifically at the tip are indicated on the corresponding bar. SFB were considered labelled if co-localization with at least 20 gold particles (black spheres) was observed, or at least 10 gold particles if labelling was restricted to the SFB tip. Individual data points corresponding to the percentages shown are in the Supplementary Data 2 file. Source data are provided as a Source Data file. The number of SFB imaged per condition is included in the graph. **f**,**g**, Immunofluorescence images of **(f)** fixed and denatured SFB incubated with a biotinylated VHH-Fc anti-Th17Ag and Streptavidin-Alexa568 and of **(g)** fixed SFB incubated with a rabbit polyclonal antibody (Ab) against the Th17Ag and a secondary antibody anti-rabbit conjugated with Alexa 568. All SFB were additionally labelled with DAPI. An extra image of a labelled IO was included as an insert delimited by a white line (f). Close-ups of the tip of an SFB filament showing Th17Ag labelling (white arrow head) were included next to the main panel. Images showing the signal from bright field (BF), Alexa-568 and corresponding merged image are shown. For all immunogold and immunofluorescence experiments, labelling was performed in two distinct days using two biological replicates for each experiment. **Scale bars**: a-d: 100 nm; f/g: 5 µm for main panel and 1 µm for inserts.

Supplementary Fig. 15

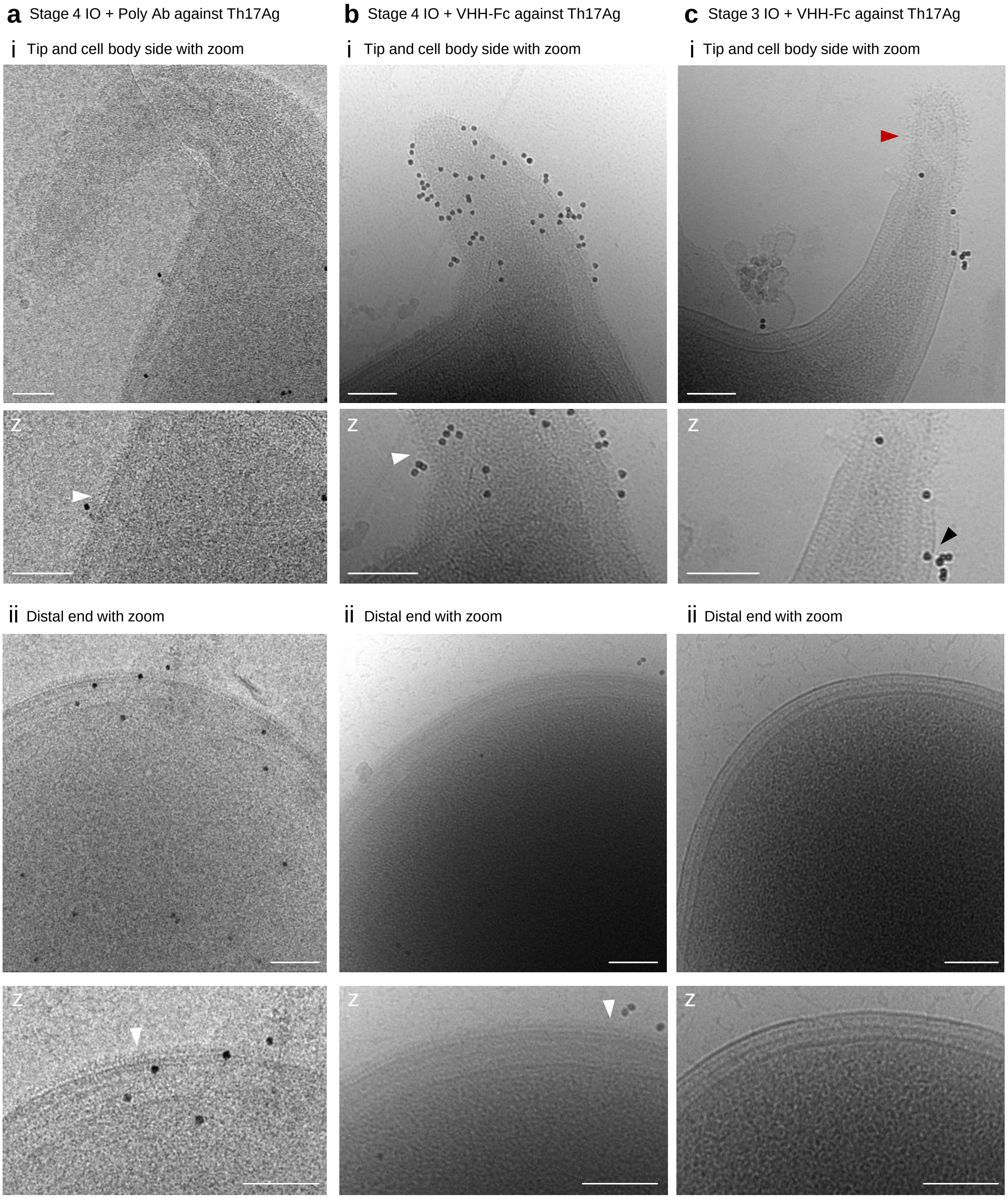

Supplementary Fig. 15. Th17Ag labelling found in Stage 4/5, Stage 4 and Stage 3 IOs. a-c, Projection images from purified and fixed SFB IOs stained using immunogold labelling. **(a)** Stage 4/5 IOs incubated with the polyclonal antibody (Poly Ab) anti-Th17Ag and showing non-tip-restricted labelling. Projection images were acquired with a Tecnai F20 electron microscope equipped with a Falcon 2 camera. **(b)** Stage 4 IOs incubated with an anti-Th17Ag nanobody fused to the Fc region of human IgG1 (VHH-Fc) showing mainly tip-restricted labelling. **(c)** Stage 3 IOs incubated with a VHH-Fc anti-Th17Ag showing labelling in regions containing an S-layer. Gold-conjugated Protein A was used for immunogold labelling. Imaging was performed at the SFB **(a-c(i))** tip/cell body side and **(a-c(ii))** distal end. Close-ups **(z)** of the SFB tip/cell body side and distal end are shown under each panel. Examples of labelled regions in which hair-like structures or the S-layer are present are shown by white and black arrow heads, respectively. A region containing unlabelled disordered hair-like structures is shown by a red arrow head. **Scale bars:** a-c: 100nm.

Supplementary Fig. 16

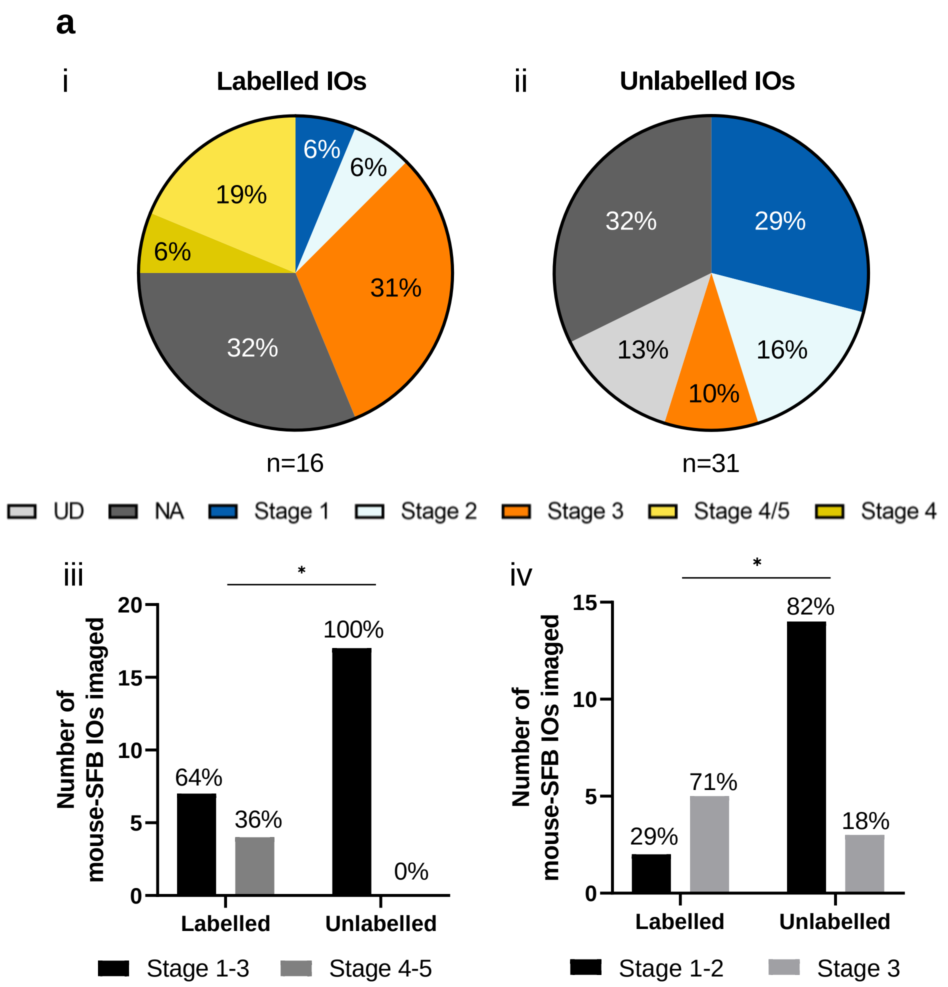

Supplementary Fig. 16. Mouse-SFB IO stages immunogold-labelled with rabbit polyclonal or VHH-Fc anti-Th17Ag. a(i-ii), Pie charts showing the percentage of each stage of IOs found **(a(i))** labelled with either rabbit polyclonal or VHH-Fc anti-Th17Ag (n=16 IOs from 4 independent experiments) and **(a(ii))** unlabeled (n=31 IOs from 4 independent experiments) when incubated with either of these antibodies. **a(iii-iv)**, Comparison of the proportions of labelled IOs assigned to **(a(iii))** early (Stages 1-3) and late SFB stages (Stages 4-5) and to **(a(iv))** Stage 3 or other early SFB stages (Stages 1-2). The statistical significance was assessed using a two-sided Fisher's exact test (a(iii): p = 0.0161; a(iv): p = 0.0207). Source data are provided as a Source Data file for all panels. The same data were used to prepare the different subpanels. NA: not assessed; ns: not significant; UD: unidentified.

Supplementary Fig. 17

a Kinetic analysis of Th17Ag binding to monovalent VHH

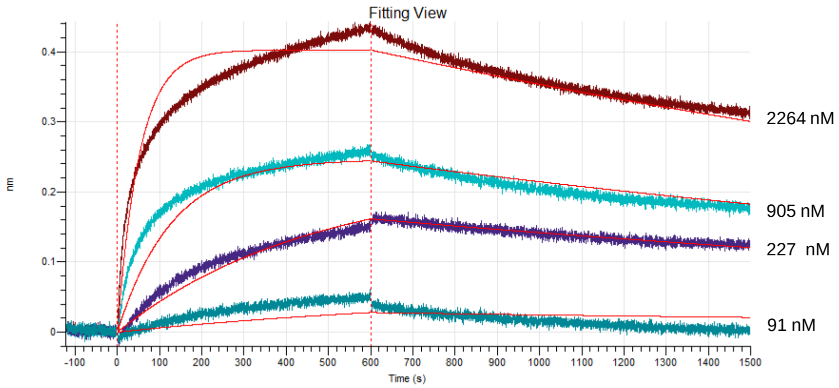

b Peptide map of the Th17Ag

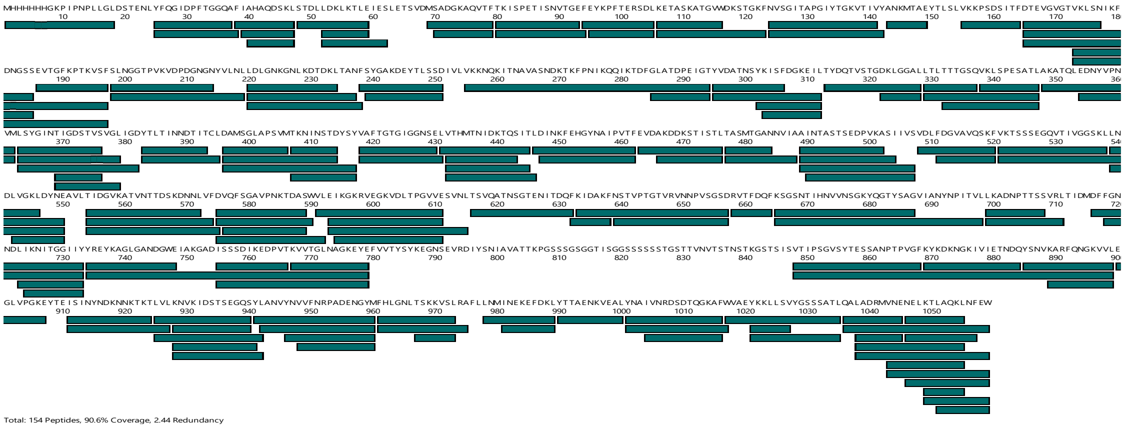

c Deuterium uptake profiles and differential uptake profile

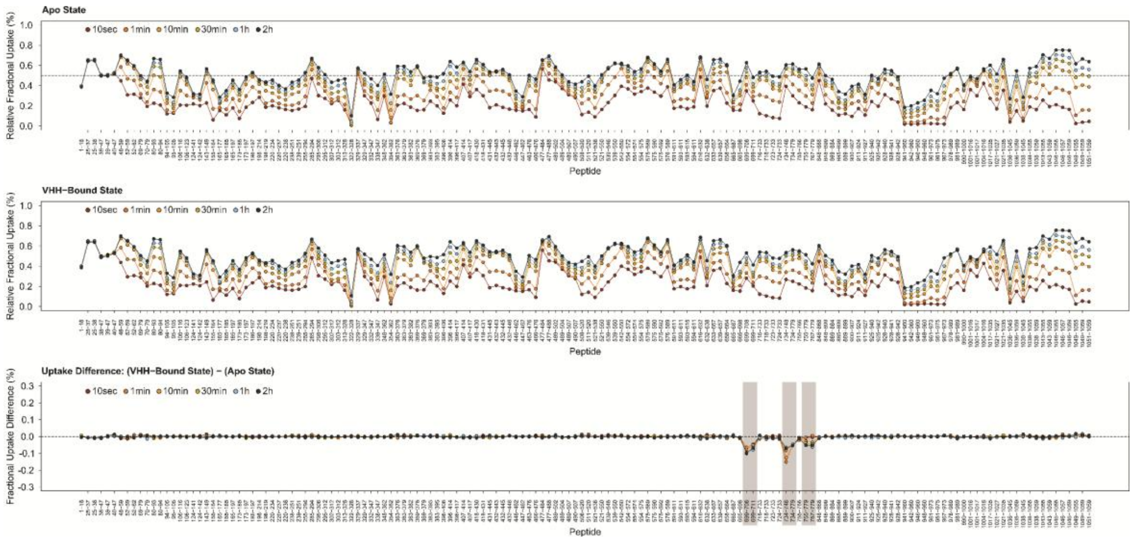

Supplementary Fig. 17. Kinetic and HDX-MS analysis of Th17Ag binding to monovalent VHH anti-Th17Ag. a, Kinetic analysis of Th17Ag binding to monovalent anti-TH17Ag nanobody (VHH). Biolayer Interferometry (BLI) was performed using an Octet HTX system. The resulting sensorgrams showing association and dissociation curves of the Th17Ag to immobilized monovalent VHH are shown. The concentrations of the Th17Ag used for the binding assays are indicated next to each curve. The fitting curves obtained with a 1:1 Langmuir model are shown in red. The kinetic analysis of Th17Ag binding to monovalent anti-TH17Ag VHH was repeated two times showing similar results. **b**, Peptide map of the Th17Ag. Each bar corresponds to a unique peptide selected for analysis by Hydrogen/ Deuterium eXchange-Mass Spectrometry (HDX-MS). A total of 154 peptides covering 90.6% of the protein sequence with a 2.44 redundancy were recovered. **c**, Effect of VHH binding on the exchange behaviour of the Th17Ag determined by HDX-MS. The deuterium uptake profiles of the Th17Ag alone (Apo State) and bound to VHH (VHH-Bound State) were used to calculate the uptake difference between the two protein states, shown by a differential fractional uptake plot. A negative value is indicative of a VHH-mediated protective effect.

Supplementary Fig. 18

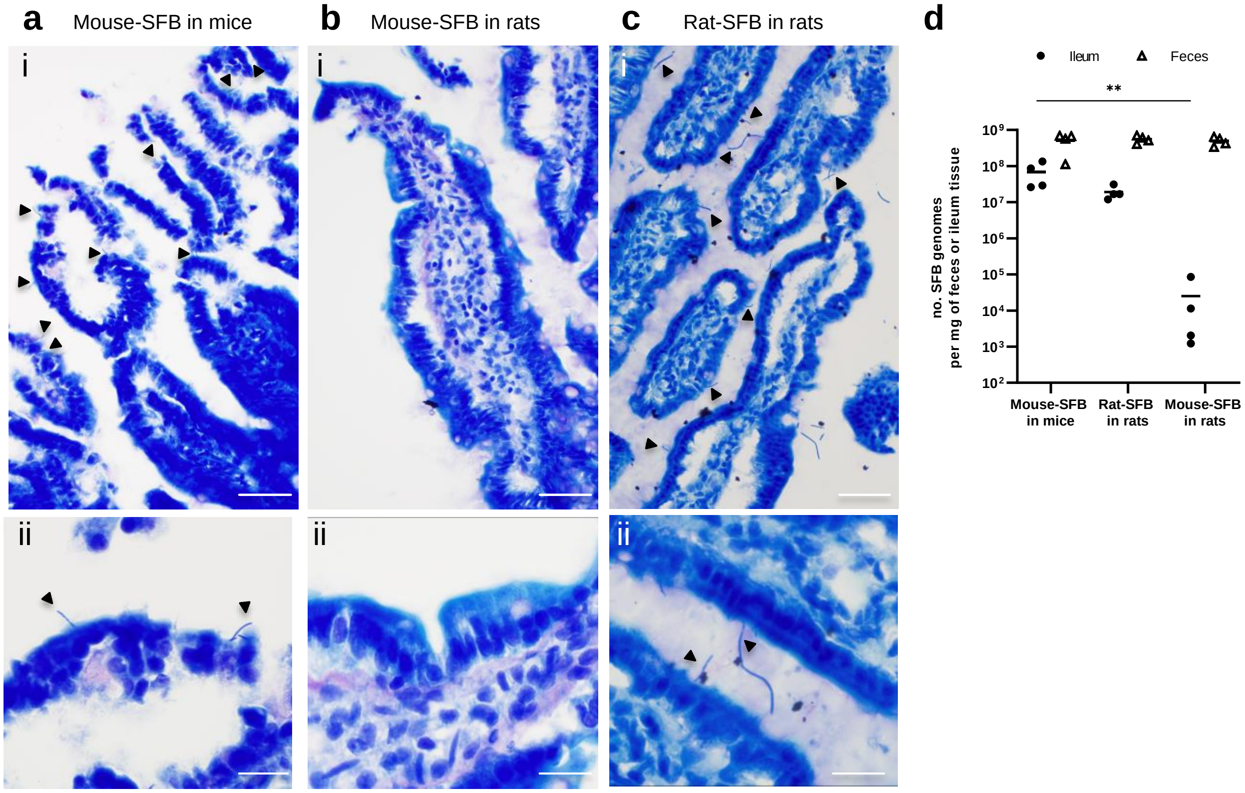

Supplementary Fig. 18. Assessment of host-specificity of SFB attachment. a-c, Giemsa stain of the terminal ileum of **(a)** mice colonized by mouse-SFB, **(b)** rats colonized by mouse-SFB, and **(c)** rats colonized by rat-SFB. **a**,**c(ii)**, Close-ups of attached SFB. **b(ii)**, Close-up of villi where no attached SFB was found. Attached SFB are highlighted with black arrow heads. **d**, SFB quantification in feces and terminal ileum biopsies of monocolonized mice and rats by qPCR. Data from 4 independent experiments and the corresponding mean are shown. The statistical significance was assessed using the Kruskal-Wallis test followed by Dunn's test correction for multiple comparisons (ileum mouse-SFB in mice vs ileum mouse-SFB in rats, p = 0.0098, the remaining comparisons were not significant). Source data are provided as a Source Data file for all panels. **Scale bars**: a-c(i): 2000 µm; a-c(ii): 50 µm.

Supplementary References

1. Hourdel, V., Volant, S., O'Brien, D. P., Chenal, A., Chamot-Rooke, J., Dillies, M. A. & Brier, S. MEMHDX: An interactive tool to expedite the statistical validation and visualization of large HDX-MS datasets. Bioinformatics 32, 3413–3419 (2016)

2. Gasteiger, E., Hoogland, C., Gattiker, A., Duvaud, S., Wilkins, M. R., Appel, R. D. & Bairoch, A. Protein Analysis Tools on the ExPASy Server. in The Proteomics Protocols Handbook Edited Protein Identification and Analysis Tools on the ExPASy Server (ed. Walker JM) (Springer Protocols Handbooks. Humana Press., 2005).
