# Supplementary material for: Segmented filamentous bacteria undergo a structural transition at their adhesive tip during unicellular to filament development
Source: Nat Commun. 2025 Dec 17;17:222. doi: 10.1038/s41467-025-66892-5 (PMC12780176; doi:10.1038/s41467-025-66892-5)
Supplement: Supplementary file 13 — Reporting Summary [file 41467_2025_66892_MOESM13_ESM.pdf]

nature portfolio

Corresponding author(s): Pamela Schnupf

Last updated by author(s): Nov 13, 2025

Reporting Summary

Nature Portfolio wishes to improve the reproducibility of the work that we publish. This form provides structure for consistency and transparency in reporting. For further information on Nature Portfolio policies, see our Editorial Policies and the Editorial Policy Checklist.

Statistics

For all statistical analyses, confirm that the following items are present in the figure legend, table legend, main text, or Methods section.

n/a	Confirmed	
☐	☒	The exact sample size (n) for each experimental group/condition, given as a discrete number and unit of measurement
☐	☒	A statement on whether measurements were taken from distinct samples or whether the same sample was measured repeatedly
☐	☒	The statistical test(s) used AND whether they are one- or two-sided<br>*Only common tests should be described solely by name; describe more complex techniques in the Methods section.*
☐	☒	A description of all covariates tested
☐	☒	A description of any assumptions or corrections, such as tests of normality and adjustment for multiple comparisons
☐	☒	A full description of the statistical parameters including central tendency (e.g. means) or other basic estimates (e.g. regression coefficient) AND variation (e.g. standard deviation) or associated estimates of uncertainty (e.g. confidence intervals)
☐	☒	For null hypothesis testing, the test statistic (e.g. F, t, r) with confidence intervals, effect sizes, degrees of freedom and P value noted<br>*Give P values as exact values whenever suitable.*
☒	☐	For Bayesian analysis, information on the choice of priors and Markov chain Monte Carlo settings
☒	☐	For hierarchical and complex designs, identification of the appropriate level for tests and full reporting of outcomes
☒	☐	Estimates of effect sizes (e.g. Cohen's d, Pearson's r), indicating how they were calculated

Our web collection on statistics for biologists contains articles on many of the points above.

Software and code

Policy information about availability of computer code

Data collection

Light Microscopy: Fluorescently-labelled bacteria were imaged using an SP8 confocal microscope (Leica) equipped with both PMTs and HyD detection systems and a LAS X acquisition software (version 3.5.7). Slides were imaged at room temperature using a HC PL APO 63x (1.40 NA) objective and z-stacks were acquired at 0.3 µm intervals with scanning at 1024 x 1024 pixels and a zoom factor of 2.5 or at 2048 x 2048 pixels and a zoom factor of 1.25. DAPI and the fluorescent dye Alexa Fluor 568 were excited using 405 nm and 561 nm lasers, respectively.
Ileal sections were imaged at room temperature using an Olympus BX53 microscope equipped with an Olympus cellSens Standard 4.2.1 software. The Olympus UPLFLN 40X objective (0.75 NA) and the UPLFLN 100X objective (1.3 NA) were used.

Cryogenic electron tomography: For the tilt series acquired at the University of Zurich, a Titan Krios microscope (Thermo Fisher Scientific) operated at 300kV equipped with a post-column energy filter (20 eV slit width, Gatan) and a K2 direct electron detector (Gatan) was used. Data acquisition was controlled by SerialEM 3.8. Tilt series were acquired at a calibrated pixel size of 1.75 or 2.21 Å using a bidirectional scheme starting at -30 ° from -60 ° to +60 ° with a 3 ° increment. For the tilt series acquired at Institut Pasteur, a 300 kV TITAN Krios electron microscope equipped with a Falcon 4i direct electron detector and a Selectris X energy filter (10 eV slit width) was used. Data acquisition was controlled by TOMO5 (version 5.14, 5.17 or 5.22, Thermo Fisher Scientific). Tilt series were acquired at a calibrated pixel size of 1.59 or 1.9 Å using a dose-symmetric scheme starting at 0 ° from -51 ° to +51 ° with a 3 ° increment. For all tilt series the projection dose was calibrated for each sample to reach a total dose of approximately 120 to 140 e/A2 and the defocus used was between -2 and -6 µm.

Cryogenic electron microscopy: Projection images were acquired using either the 300kV Titan Krios from Institut Pasteur (mentioned above) or a 200 kV Tecnai F20 (Thermo Fisher Scientific) equipped with a direct detector Falcon II (Thermo Fisher Scientific). Projection image acquisition was performed using the software EPU (Thermo Fisher Scientific), when using the Tecnai F20.

Protein purification: Th17Ag purification was performed using an AKTA-FPLC purification system equipped with a UNICORN 5.31 following the instructions of the manufacturer. Nanobody purification was performed using an AKTA-Start purification system equipped with a UNICORN start 3.1 according to the manufacturer instructions.

Assessment of antibody binding to Th17Ag: Binding was assessed by ELISA. The absorbance was measured at 415 nm using an iMark Microplate Absorbance Reader (BioRad) equipped with an MPM6 software (Version 6.3, BioRad).

Binding kinetics of VHH anti-Th17Ag: The binding kinetics of the VHH anti-Th17Ag VHH were obtained by Biolayer Interferometry (BLI) using an Octet HTX system (Sartorius) equipped with an Octet Data Acquisition software version 13.0 (Sartorius) at 30°C.

Data analysis

Data was plotted and statistical analysis was performed using GraphPad Prism 9.10 (GraphPad Software Inc).

Light microscopy: The contrast of representative images was adjusted in Fiji version 2.14.0 (RRID:SCR_002285) for visualization purposes.

Cryogenic electron tomography: Cryo-ET data analysis was performed using the IMOD software package version 4.11 (RRID:SCR_003297). For each tilt series acquired, frame alignment was performed using either MotionCor2 1.6.4 or the Alignframes program from IMOD. IMOD was also used for tilt alignment using fiducial markers and tomogram reconstruction was performed with an antialiasing filter using dose weighting and a SIRT-like filter with 10-20 iterations. For tomograms selected as representative, fiducials were erased with the IMOD findbeads3d tool. These tomograms were further subjected to topaz denoising using a default 10 Å/px pre-trained model, for visualization purposes. Segmentation masks for representative IMOD models were prepared in UCSF ChimeraX version 1.7 (RRID:SCR_015872).

Cryogenic electron microscopy: The contrast of projection images selected as representative was adjusted and a Gaussian low-pass filter with 0.5 to 1 px radius was applied using Fiji's unsharped mask tool when necessary for visualization purposes. The ccderaser function of IMOD was used to erase the black pixel located at the center of the original images from immunogold labelled samples acquired with the 200 kV Tecnai F20.

HDX-MS: The initial peptide map of the Th17Ag was generated by database searching in ProteinLynX Global server 3.0 (Waters corporation). The peptide map was refined in DynamX 3.0 (Waters corporation). The MEMHDX software (version 0.01) was used to statistically validate HDX-MS datasets.

Binding kinetics of VHH anti-Th17Ag: Raw data was processed using Octet Data Analysis Studio (version 13.0) and the data were fitted to a 1:1 Langmuir binding model.

For manuscripts utilizing custom algorithms or software that are central to the research but not yet described in published literature, software must be made available to editors and reviewers. We strongly encourage code deposition in a community repository (e.g. GitHub). See the Nature Portfolio guidelines for submitting code & software for further information.

Data

Policy information about availability of data

All manuscripts must include a data availability statement. This statement should provide the following information, where applicable:

- Accession codes, unique identifiers, or web links for publicly available datasets
- A description of any restrictions on data availability
- For clinical datasets or third party data, please ensure that the statement adheres to our policy

Reconstructed tomograms used as representative have been deposited in the EMDB under the following accession codes: EMD-52655; EMD-52667; EMD-52668; EMD-52669; EMD-52670; EMD-52671; EMD-52673; EMD-52674; EMD-52675; EMD-52676; EMD-52677; EMD-52678; EMD-52679; EMD-52680; EMD-52682; EMD-52683; EMD-52684; EMD-52685; EMD-52687; EMD-52688; EMD-52689; EMD-52690; EMD-52691; EMD-52692; EMD-52693; EMD-52694; EMD-52695; EMD-52696; EMD-52697; EMD-52698; EMD-52699; EMD-52700; EMD-52701; EMD-52702; EMD-52703; EMD-52856; EMD-54603; EMD-54605; EMD-54607; EMD-54608. The EMDB accession codes for reconstructed tomograms were indicated in the figures which include their corresponding tomographic slices. The mass spectrometry data have been deposited in the ProteomeXchange Consortium via the PRIDE61 partner repository under the accession code PXD060041. The characteristics of all the bacteria included in the cryo-EM/cryo-ET dataset and the immunogold labelling dataset are included in Supplementary Data 1 and Supplementary Data 2, respectively. Given the large size of the dataset, the remaining data, including cryo-EM, cryo-ET and confocal microscopy images, and newly generated materials are available upon request to the corresponding author. Source Data are provided with this paper.

Research involving human participants, their data, or biological material

Policy information about studies with human participants or human data. See also policy information about sex, gender (identity/presentation), and sexual orientation and race, ethnicity and racism.

Reporting on sex and gender	Our studies involve no human participants, their data, or biological material.
Reporting on race, ethnicity, or other socially relevant groupings	Our studies involve no human participants, their data, or biological material.
Population characteristics	Our studies involve no human participants, their data, or biological material.
Recruitment	Our studies involve no human participants, their data, or biological material.
Ethics oversight	Our studies involve no human participants, their data, or biological material.

Note that full information on the approval of the study protocol must also be provided in the manuscript.

Field-specific reporting

Please select the one below that is the best fit for your research. If you are not sure, read the appropriate sections before making your selection.

[x] Life sciences [ ] Behavioural & social sciences [ ] Ecological, evolutionary & environmental sciences

For a reference copy of the document with all sections, see nature.com/documents/nr-reporting-summary-flat.pdf

Life sciences study design

All studies must disclose on these points even when the disclosure is negative.

Sample size	For the cryo-EM/ET dataset, we purified SFB from at least 4 rodents in different days (having at least 4 biological replicates) and imaged bacteria during multiple microscopy sessions. The selected sample sizes were found adequate upon statistical analysis of the parameters of interest, and we confirmed that comparable morphological results were found in different replicates. For other experiments, we selected the sample sizes based on standards of the field, using generally n=3 and at least n=2. We found these sample sizes adequate since comparable results were obtained for different replicates.
Data exclusions	No data was excluded from the analyses.
Replication	All attempts of replication were successful. Experiments were performed at least two times showing comparable results.
Randomization	Germ-free mice with ages between 7 and 13 weeks and germ-free rats with ages between 7 and 17 weeks were selected based on availability and used to establish a monocolonization with either mouse-SFB or rat-SFB. Allocation of animals to experimental groups was random. The bacteria identified during the cryogenic electron microscopy sessions were imaged and subsequently taken into consideration in the analyses reported if the features of interest could be visualized.
Blinding	The different phenotypes reported were identified independently by different researchers to avoid subjectivity. All measurements were quantitative and the regions/features to measure were clearly defined to avoid individual bias.

Reporting for specific materials, systems and methods

We require information from authors about some types of materials, experimental systems and methods used in many studies. Here, indicate whether each material, system or method listed is relevant to your study. If you are not sure if a list item applies to your research, read the appropriate section before selecting a response.

Materials & experimental systems

n/a	Involved in the study	
[ ]	[x]	Antibodies
[ ]	[x]	Eukaryotic cell lines
[x]	[ ]	Palaeontology and archaeology
[ ]	[x]	Animals and other organisms
[x]	[ ]	Clinical data
[x]	[ ]	Dual use research of concern
[x]	[ ]	Plants

Methods

n/a	Involved in the study	
[x]	[ ]	ChIP-seq
[x]	[ ]	Flow cytometry
[x]	[ ]	MRI-based neuroimaging

Antibodies

Antibodies used	The following antibodies were used for ELISA, immunofluorescence and immunogold labelling: rabbit polyclonal antibody anti-SFBNYU_003340 (1:100, Yang et al. 2014, 10.1038/nature13279) and VHH-Fc anti-AID45212 (10 µg/mL, this study).<br><br>The goat polyclonal anti-rabbit IgG (H+L) conjugated with Alexa Fluor 568 (1:500, Thermo Fisher Scientific, A-11036, RRID: AB_10563566) was used as a secondary antibody for the immunofluorescence.<br><br>The following antibodies were used for the detection of binding to the purified protein AID45212 by ELISA: mouse monoclonal antibody anti-c-Myc (1:500, Bio-Techne, #NB600-302), goat polyclonal anti-mouse IgG (whole molecule) conjugated with alkaline phosphatase (1:10000, Sigma, A9316, RRID: AB_258446), mouse monoclonal anti-human IgG1 Fc secondary antibody conjugated with HRP (1:1000, Thermo Fisher Scientific, A-10648, RRID: AB_2534051) and goat anti-rabbit IgG (whole molecule) conjugated with alkaline phosphatase (1:10000, Sigma, A3687, RRID: AB_258103).<br><br>The mouse monoclonal antibody anti-M13 bacteriophage conjugated with HRP (1:2500, Abcam, ab305291) was used for phage-ELISA.<br><br>The following antibodies were used for western blot: mouse monoclonal antibody anti-polyHistidine conjugated with peroxidase (1:5000, Sigma, A7058, RRID:AB_258326) and horse anti-mouse IgG conjugated with HRP (1:20000, Cell Signaling, #7076).
Validation	The binding of the rabbit polyclonal antibody anti-SFBNYU_003340 to the protein SFBNYU_003340 (Th17Ag) was verified by Ladinsky et al. 2019 (10.1126/science.aat4042). We have also confirmed the binding of both the rabbit polyclonal antibody anti-SFBNYU_003340 and the VHH-Fc anti-AID45212 to the protein AID45212 (homolog of SFBNYU_003340 in mouse-SFB-NL, the strain used for this study) by ELISA (Supplementary Fig. 12c). Please refer to the manufacturers' websites to access the validation results of the commercially available antibodies used in this study.

Eukaryotic cell lines

Policy information about cell lines and Sex and Gender in Research

Cell line source(s)	The Expi293 cells used in this study were obtained from Thermo Fisher Scientific (Cat. No. A14527). These cells are a derivative of the HEK293F cell line, optimized for high-yield transient expression in suspension culture. The TC7 cells used to co-culture SFB for subsequent immunization of an alpaca to generate nanobodies against SFB proteins were provided by Alain Servin, Faculté de Pharmacie de Chatenay Malabry.
Authentication	The Expi293 cell line was purchased directly from the manufacturer, and no additional authentication was required as it is a well-characterized, commercially distributed line. The TC7 cell line was not authenticated.
Mycoplasma contamination	Routine mycoplasma contamination checks were not performed. Nevertheless, cell viability and protein expression levels remained consistent throughout all experiments, indicating that the cultures were stable and healthy. The TC7 cell line routinely tested negative for mycoplasma contamination using a mycoplasma detection kit (MycoStrip rep-mys-20, InvivoGen).
Commonly misidentified lines<br>(See ICLAC register)	To our knowledge, Expi293 and TC7 cells are not among the commonly misidentified cell lines. The identity of Expi293 cells is well established through the supplier's quality control process.

Animals and other research organisms

Policy information about studies involving animals; ARRIVE guidelines recommended for reporting animal research, and Sex and Gender in Research

Laboratory animals	Germ-free C57BL/6J mice aged between 7 and 13 weeks and germ-free rats F344 aged between 7 and 17 weeks were monocolonized with SFB. One adult male alpaca (Lama pacos) was immunized with purified SFB for nanobody generation against SFB proteins.
Wild animals	No wild animals were used in this study.
Reporting on sex	Both male and female mice were used. These were selected based on availability and regardless of their sex. Only male rats were used. Animal sex was not considered in the study design since SFB colonization was the only parameter of interest and no other host parameter was recorded. The immunized alpaca was a male.
Field-collected samples	No field-collected samples were used in this study.
Ethics oversight	Rodent (dap210054 and dap220096) and alpaca (2020-27412) experiments were approved by the Institut Pasteur ethical committee for animal experimentation (Comité d'Ethique en Expérimentation Animale, CETEA, registry number #89) and authorized by the Ministère de l'Enseignement Supérieur, de la Recherche et de l'Innovation. Animals were housed either at the animal facility of Institut Pasteur (IP) (agreement number 75-15-01) or at the animal facility of SFR Necker (agreement number A75-15-34).

Note that full information on the approval of the study protocol must also be provided in the manuscript.

Plants

Seed stocks

Report on the source of all seed stocks or other plant material used. If applicable, state the seed stock centre and catalogue number. If plant specimens were collected from the field, describe the collection location, date and sampling procedures.

Novel plant genotypes

Describe the methods by which all novel plant genotypes were produced. This includes those generated by transgenic approaches, gene editing, chemical/radiation-based mutagenesis and hybridization. For transgenic lines, describe the transformation method, the number of independent lines analyzed and the generation upon which experiments were performed. For gene-edited lines, describe the editor used, the endogenous sequence targeted for editing, the targeting guide RNA sequence (if applicable) and how the editor was applied.

Authentication

Describe any authentication procedures for each seed stock used or novel genotype generated. Describe any experiments used to assess the effect of a mutation and, where applicable, how potential secondary effects (e.g. second site T-DNA insertions, mosiacism, off-target gene editing) were examined.
