# Supplementary material for: Segmented filamentous bacteria undergo a structural transition at their adhesive tip during unicellular to filament development
Source: Nat Commun. 2025 Dec 17;17:222. doi: 10.1038/s41467-025-66892-5 (PMC12780176; doi:10.1038/s41467-025-66892-5)
Supplement: Supplementary file 14 — Description of Additional Supplementary Files [file 41467_2025_66892_MOESM14_ESM.pdf]

Description of Additional Supplementary Files:

Supplementary Data 1: Characteristics of all SFB analyzed in the cryo-EM/cryo-ET dataset. HLL: hairlike layer, NA: not assessed, UD: unidentified.

Supplementary Data 2: Characteristics of all SFB analyzed after immunogold labelling. NA: not assessed, UD: unidentified.

Supplementary Movie 1: Representative tomogram of a mouse-SFB IO tip (EMD-52655) with the corresponding segmentation showing the Slayer, cell wall, membrane, flagella, intracellular vesicles and tracks. Scale bar: 50nm.

Supplementary Movie 2: Representative tomogram of a mouse-SFB IO tip (EMD-52667) with the corresponding segmentation showing the Slayer, cell wall, membrane and chemosensory array. Scale bar: 50nm.

Supplementary Movie 3: Representative tomogram of a mouse-SFB IO tip (EMD-52668) with the corresponding segmentation showing the Slayer, cell wall, membrane and representative intracellular filaments. Scale bar: 50nm.

Supplementary Movie 4: Representative tomogram of a mouse-SFB IO tip (EMD-52669) with the corresponding segmentation showing the Slayer, cell wall, membrane and representative platelike structures. Scale bar: 50nm.

Supplementary Movie 5: Representative tomogram of a Stage 1 mouse-SFB IO tip (EMD52685) with the corresponding segmentation showing the S-layer, cell wall and membrane. Scale bar: 50nm.

Supplementary Movie 6: Representative tomogram of a Stage 3 mouse-SFB IO tip (EMD52676) with the corresponding segmentation showing the S-layer, cell wall, membrane and disordered hair-like structures (disHLS). Scale bar: 50nm.

Supplementary Movie 7: Representative tomogram of a Stage 4 mouse-SFB filament tip (EMD-52677) with the corresponding segmentation showing the hair-like layer (HLL), cell wall, membrane, the individual disordered (disHLS) and ordered hair-like structures (ordHLS). Scale bar: 50nm.

Supplementary Movie 8: Representative tomogram of a Stage 5 mouse-SFB filament tip (EMD-52678) with the corresponding segmentation showing the hair-like layer (HLL), cell wall, membrane and individual ordered hair-like structures (ordHLS). Scale bar: 50nm.

Supplementary Movie 9: Representative tomogram of a transitional Stage 3 rat-SFB IO tip (EMD-54603) with the corresponding segmentation showing the hair-like layer (HLL), S-layer, cell wall, membrane, individual disordered (disHLS) and ordered hair-like structures (ordHLS). Scale bar: 50nm.
